# *Caenorhabditis elegans* Cyclin B3 Is Required for Multiple Mitotic Processes Including Alleviation of a Spindle Checkpoint–Dependent Block in Anaphase Chromosome Segregation

**DOI:** 10.1371/journal.pgen.1001218

**Published:** 2010-11-24

**Authors:** Gary M. R. Deyter, Tokiko Furuta, Yasuhiro Kurasawa, Jill M. Schumacher

**Affiliations:** 1 Department of Genetics, University of Texas M. D. Anderson Cancer Center, Houston, Texas, United States of America; 2 Program in Genes and Development, University of Texas Graduate School of Biomedical Sciences, Houston, Texas, United States of America; National Institute of Diabetes and Digestive and Kidney Diseases, United States of America

## Abstract

The master regulators of the cell cycle are cyclin-dependent kinases (Cdks), which influence the function of a myriad of proteins via phosphorylation. Mitotic Cdk1 is activated by A-type, as well as B1- and B2-type, cyclins. However, the role of a third, conserved cyclin B family member, cyclin B3, is less well defined. Here, we show that *Caenorhabditis elegans* CYB-3 has essential and distinct functions from cyclin B1 and B2 in the early embryo. CYB-3 is required for the timely execution of a number of cell cycle events including completion of the MII meiotic division of the oocyte nucleus, pronuclear migration, centrosome maturation, mitotic chromosome condensation and congression, and, most strikingly, progression through the metaphase-to-anaphase transition. Our experiments reveal that the extended metaphase delay in CYB-3–depleted embryos is dependent on an intact spindle assembly checkpoint (SAC) and results in salient defects in the architecture of holocentric metaphase chromosomes. Furthermore, genetically increasing or decreasing dynein activity results in the respective suppression or enhancement of CYB-3–dependent defects in cell cycle progression. Altogether, these data reveal that CYB-3 plays a unique, essential role in the cell cycle including promoting mitotic dynein functionality and alleviation of a SAC–dependent block in anaphase chromosome segregation.

## Author Summary

Every time a cell divides in two, the genetic material, DNA, is copied; each copied chromosome is referred to as a pair of sister chromatids. Each chromatid must be cleanly separated from its sister so that each daughter cell inherits the same DNA complement as the starting cell. The mitotic spindle is a cellular machine that physically separates the sister chromatids from one another. The chromatids are attached to the spindle at kinetochores, which are structures built at specific sites (centromeres) on each chromatid. The cell monitors the attachment of each chromatid and blocks their separation until they are all properly attached. This process is called the spindle assembly checkpoint (SAC). Here we report that loss of an evolutionarily conserved cell cycle regulator, Cyclin B3/CYB-3, results in an unusual and strikingly persistent SAC–dependent delay in sister chromatid separation. Furthermore, CYB-3 promotes the activity of a cellular motor, dynein, in this and other mitotic processes. Altogether, our results indicate that Cyclin B3 genetically interacts with mitotic dynein and is absolutely required to satisfy a SAC–dependent inhibition in sister chromatid separation.

## Introduction

The eukaryotic cell cycle is driven by the temporally controlled activation of cyclin-dependent kinases (CDKs) in association with their requisite cofactors, the cyclins [1]. The expression and stability of individual cyclins is coordinated with specific cell cycle stages. For instance, cyclin E is expressed as cells enter G1 and is degraded in early S phase, while cyclin B levels rise in G2 and fall at the metaphase-to-anaphase transition [1]. Cyclins not only contribute to the temporal activation of specific CDKs at particular cell cycle transitions, but also appear to provide substrate specificity [2].

As cells prepare to enter mitosis, cyclin B/Cdk1 complexes phosphorylate a host of substrates leading to chromosome condensation, centrosome maturation, and nuclear envelope breakdown [3]. During this period, the chromosome/microtubule interface, the kinetochore, is constructed from several protein complexes that are coordinately built at the centromere, an epigenetically defined chromosomal location [4]. In budding yeast, the centromere consists of a defined 125 base-pair sequence, while in fission yeast and higher eukaryotes centromeres are heterochromatin rich and are not identified by specific nucleotide sequences. Other organisms, including *C. elegans*, have holocentric chromosomes with kinetochores along their entire length [5]. Despite these differences, all eukaryotic centromeres harbor specialized nucleosomes wherein the canonical histone H3 is replaced by the centromere-specific histone CENP-A/CenH3 [6].

The *raison d'être* for mitosis is the equal partitioning of replicated genetic material to each daughter cell. Hence, progression through mitosis is inextricably linked to the state of kinetochore-microtubule attachment. To be properly segregated, each pair of sister chromatids must be attached to the mitotic spindle in a bipolar fashion [7]. Once bipolar attachment is achieved, the cohesed sister centromeres and kinetochores are under tension; stretching occurs between sister centromeres and within kinetochores [8]. The spindle assembly checkpoint (SAC) monitors this process and is exquisitely sensitive to the attachment and tension state of individual kinetochores. The SAC delays the metaphase-anaphase transition via inhibition of the anaphase-promoting complex (APC) until all chromosomes are attached and are under tension. The SAC consists of several components, including the Bub- and Mad-related proteins first identified in genetic screens in budding yeast, and is influenced by the Mps1, Polo, and Aurora B kinases [7]. Unattached kinetochores recruit Mad2 [9], [10], while the Polo and Aurora B kinases monitor tension [11], [12]. Aurora B is localized to the inner centromere where it destabilizes inappropriate kinetochore-microtubule interactions via phosphorylation of microtubule-associated proteins, including Ndc80/Hec1, MCAK, and Kif2 [13]–[16]. This activity releases kinetochore-microtubules, resulting in “free” kinetochores that can undergo reattachment [17].

It has become increasingly clear that once a cell engages a checkpoint such as the SAC, the checkpoint must be shut-off or silenced once the checkpoint is satisfied (*i.e.*, all chromosomes are attached and under tension) [18]. Inter-centromeric and intra-kinetochore stretching resulting from bipolar attachment appears to limit the interaction between Aurora B and its substrates at the outer kinetochore, resulting in the stabilization of bipolar attachments [19]. In addition, the minus-end directed protein dynein is required for SAC silencing as it strips Mad2 and other checkpoint proteins from kinetochores and traffics them along kinetochore-microtubules to centrosomes [20]–[23]. When dynein function is compromised, the APC remains inhibited and the metaphase-to-anaphase transition is delayed, even when all chromosomes are properly attached.

A key target of the APC is cyclin B, a mitotic-specific Cdk1 partner. Mammals have three B-type cyclins -B1, B2, and B3- which appear to have both overlapping and specific functions [24]. While cyclins B1 and B2 are highly similar, B3 forms a distinct sub-family with more sequence conservation among B3 proteins from divergent species than with B1 and B2 cyclins from the same species [25]. While human B1 and B2 cyclins are highly expressed in dividing cells, B3 is found at much lower levels [24]. However, human B3 is also highly expressed in male and female meiotic germ cells [25], [26]. In *Drosophila*, a cyclin B3/CycB3 mutant is female sterile yet viable [27]. RNAi experiments also revealed that cycB3 is not essential for mitosis, but does share a partially redundant function with cycB to promote timely anaphase entry [28]. To date, a specific, functional role for cyclin B3 in mitosis has not been revealed.

*C. elegans* harbors four partially redundant cyclin B family members [29], [30]. While previous studies revealed a role for CYB-3 in progression through meiosis II and the oocyte-embryo transition [31]–[33], here we demonstrate that loss of CYB-3 leads to specific defects in multiple dynein-related mitotic processes. Strikingly, CYB-3 depletion leads to an unprecedented *C. elegans* mitotic phenotype: a persistent block in the initiation of anaphase chromosome segregation. The experiments herein reveal the nature of this phenotype and lead to a working model whereby CYB-3 genetically promotes mitotic dynein functionality and is required to satisfy the spindle assembly checkpoint.

## Results

### CYB-3–depleted embryos exhibit defects in MII, pronuclear migration, and synchronous mitotic entry

The first mitotic division of *C. elegans* embryogenesis occurs after fertilization and the completion of the meiotic divisions of the oocyte nucleus. Upon extrusion of the second polar body, the maternal pronucleus migrates towards the paternal pronucleus at the posterior end of the embryo. As their chromosomes condense, the two pronuclei join and traverse toward the center of the embryo while the growing mitotic spindle undergoes a rotation to align with the long axis. Nuclear envelope breakdown and microtubule attachment ensue, culminating with chromosome alignment at the metaphase plate followed by immediate anaphase sister chromatid segregation, cleavage furrow ingression, and mitotic exit [34].

To assess the role of *C. elegans* CYB-3 in these processes, young hermaphrodites (L4 larvae) were fed bacteria expressing *cyb-3* dsRNA. This RNAi treatment resulted in efficient CYB-3 depletion (Figure S1A and Text S1) and fully penetrant embryonic lethality. To fully address this phenotype, progression through the meiotic divisions and early embryogenesis were monitored by live imaging of fertilized oocytes and embryos expressing either GFP::Histone H2B; GFP::γ-tubulin (TH32) [35] or mCherry::Histone H2B; GFP::α-tubulin (OD57) [36] to visualize chromosomes, centrosomes, and/or spindle microtubules. As in controls, the maternal nucleus of newly fertilized *cyb-3(RNAi)* oocytes underwent an apparently normal first meiotic division followed by extrusion of the first polar body at the anterior end of the embryo (Videos S1, S2). Likewise, both types of embryos generated a second meiotic spindle with chromosomes aligned at the metaphase plate. However, in the majority of *cyb-3(RNAi)* embryos, anaphase II did not occur. Sister chromatids failed to separate from one another and a second polar body was not extruded (Videos S1, S2); similar findings were recently reported [30]. In many of these embryos, the meiotic spindle “floated” away from the anterior cortex and ultimately disassembled in the anterior third of the embryo (Video S2). This MII defect resulted in either multiple maternal pronuclei or a single diploid pronucleus (Videos S3, S4, S5, S6).

**Figure 1:**
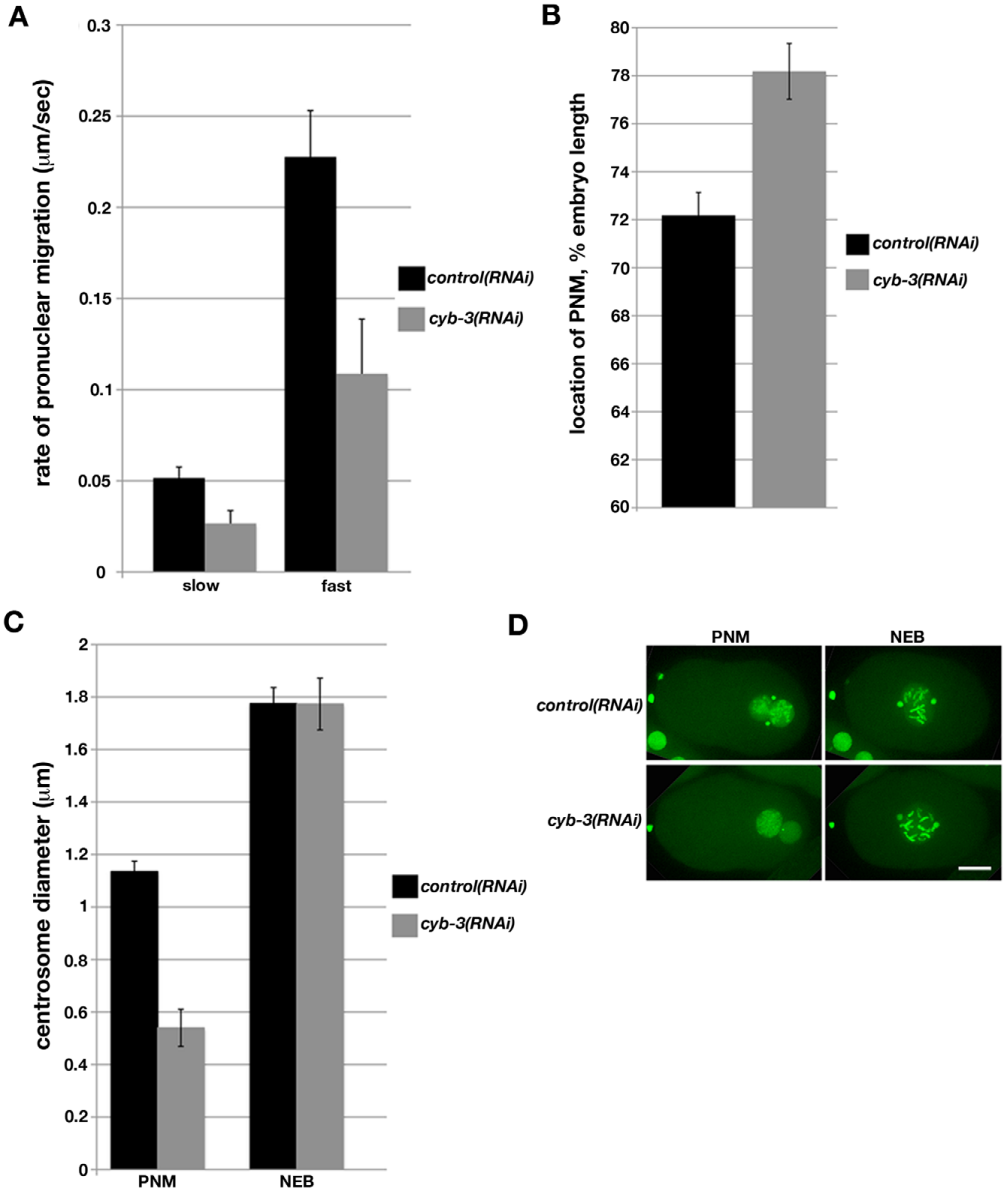
Depletion of *C. elegans* Cyclin B3 leads to defects in pronuclear migration and synchrony of chromosome condensation. Embryos from TH32 (GFP::Histone H2B;GFP::γ-tubulin) and OD57 (mCherry::Histone H2B; GFP:: α-tubulin) hermaphrodites fed *control* or *cyb-3* dsRNA were subjected to live imaging. A) The rate of pronuclear migration was calculated by measuring the distance (µm) between the maternal and paternal pronuclei with respect to time (seconds). Slow: slow phase (0–40% EL); Fast: fast phase (>40% EL); 0%: anterior end; *control(RNAi)*, n = 5; *cyb-3(RNAi)*, n = 7; Error bars: standard error of the means (SEM). B) The position of PNM was measured as the distance from the position of PNM to the anterior end and is displayed as % EL. *control(RNAi)*, n = 12; *cyb-3(RNAi)*, n = 14; Error bars: SEM; p = 0.0007. C) Centrosome size in TH32 embryos treated with *control* and *cyb-3(RNAi)* was measured at PNM and NEB. n =  centrosomes; *control(RNAi)*, PNM: n = 17, NEB: n = 17; *cyb-3(RNAi)*, PNM: n = 11, NEB: n = 13; Error bars: SEM; PNM: p<0.0001; NEB: p = 0.9. D) Selected images from Videos S3 and S4: TH32 embryos treated with *control* and *cyb-3(RNAi)* at PNM and NEB. Anterior is to the left in all images. Scale bar: 10 µm.

Upon completion of the two meiotic divisions in wild-type cells, the maternal pronucleus migrates toward the male pronucleus, which is positioned at the posterior end of the embryo. The maternal pronucleus migrates in two distinct phases, with an initial slow velocity until it reaches approximately 40% of embryo length (EL) from the anterior end (Anterior: 0%; Posterior: 100%) [22], [37]. The migration rate then increases significantly (fast phase). Since the paternal pronucleus migrates toward the anterior, the two pronuclei meet at approximately 70% of EL [37]. Compared to control, both phases of maternal pronuclear migration were approximately two-fold slower in *cyb-3(RNAi)* embryos (Figure 1A; Videos S3, S4, S5, S6). In addition, paternal pronuclear migration toward the anterior was greatly reduced, resulting in pronuclear meeting (PNM) occurring significantly closer to the embryo posterior (Figure 1B, 1D, and Figure 3C; Videos S4, S6).

**Figure 2:**
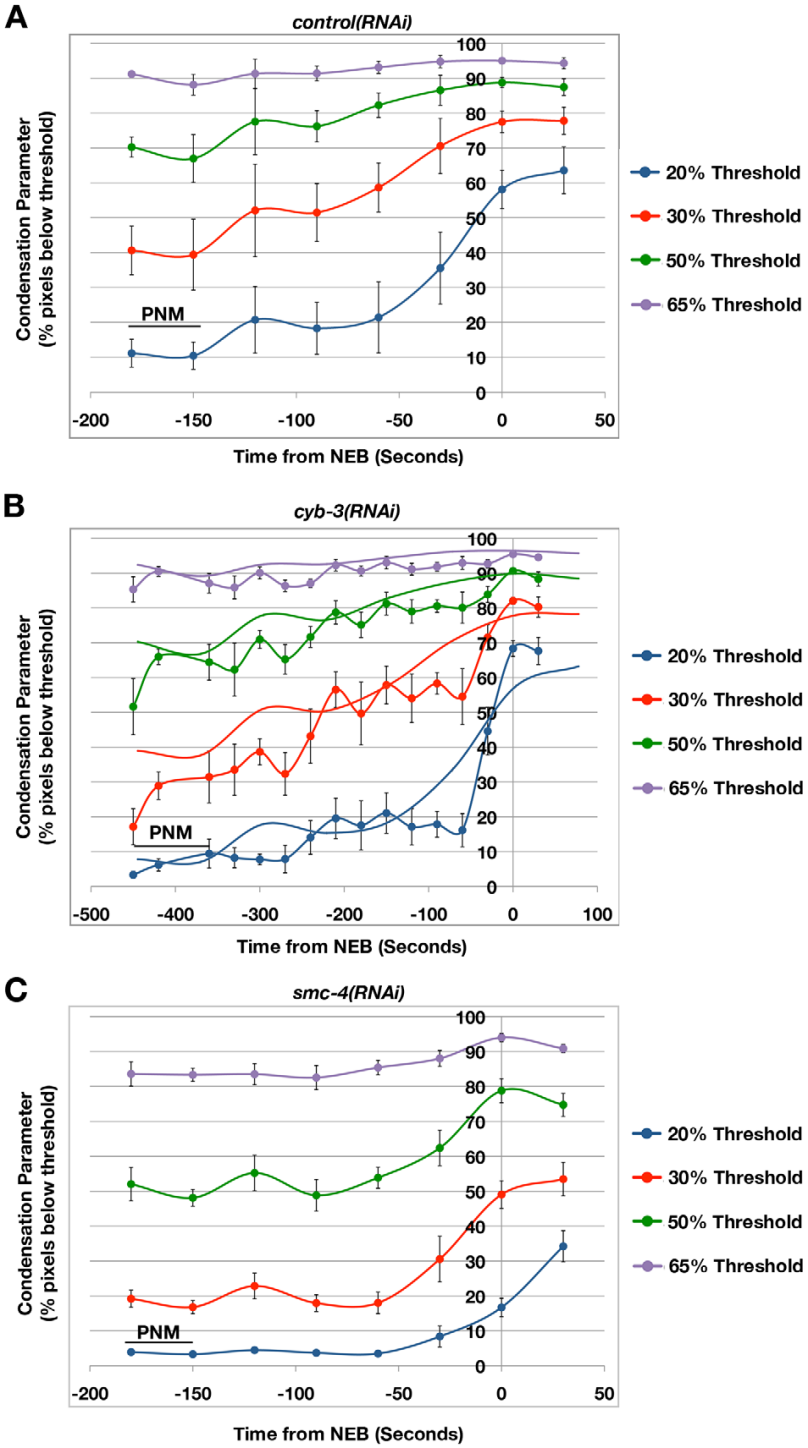
Mitotic chromosome condensation is delayed in *cyb-3(RNAi)* embryos. Embryos from TH32 (GFP::Histone H2B;GFP::γ-tubulin) hermaphrodites treated with A) *control(RNAi)*, B) *cyb-3(RNAi)*, or C) *smc-4(RNAi)* were subjected to live imaging. Condensation of the paternal pronucleus was measured as described in Materials and Methods. Condensation parameters (% pixels below threshold) are plotted for four thresholds (20, 35, 50, and 65%) with respect to time from NEB (t = 0) [87]. PNM: Pronuclear meeting. SMC-4-depleted embryos were used as a control for loss of condensin complexes and mitotic chromosome condensation [51]. The *control(RNAi)* results are overlaid on the *cyb-3(RNAi)* panel to assist in direct comparison. n =  embryos; *control(RNAi)*, n = 6; *cyb-3(RNAi)*, n = 7; *smc-4(RNAi)*, n = 8; Error bars: SEM.

*C. elegans* oocytes are devoid of centrioles and centrosomes [38]. Therefore, the centriole donated by the sperm is the sole mitotic organizing center (MTOC) in the newly fertilized one-cell embryo [39]. The paternal centriole duplicates upon completion of the meiotic divisions of the oocyte nucleus. As the maternal pronucleus migrates, the centrioles recruit pericentriolar material and separate away from one another along the surface of the paternal pronucleus. Concurrently, condensation of the maternal and paternal pronuclei occurs in a synchronous manner. We noted that the maturing centrosomes in *cyb-3(RNAi)* embryos were much smaller compared to controls. At the time of PNM, *cyb-3(RNAi)* centrosomes were approximately two-fold smaller than control centrosomes (Figure 1C, 1D; Videos S3, S4). However, by nuclear envelope breakdown (NEB), there was no appreciable difference in centrosome size between CYB-3-depleted embryos and controls. Curiously, condensation of the paternal and maternal pronuclei was asynchronous in *cyb-3(RNAi)* embryos; condensation of the paternal pronucleus was significantly delayed with respect to the maternal pronucleus (Figure 1D; Videos S3, S4). However, as with centrosome size, condensation of the paternal pronucleus also “caught up” to control levels by NEB (Figure 1D, Figure 2, and Figure S2; Videos S3, S4). These defects are not likely to be secondary consequences of a failure to undergo MII anaphase since other MII defective mutants do not display these phenotypes [40].

Asynchrony of pronuclear condensation is a feature of mutants that fail to undergo pronuclear migration [37]. However, it is the maternal pronucleus that is delayed in these cases. This delay is thought to be due to the increased distance between a stationary maternal pronucleus and centrosome-based signals that promote mitotic entry [41], [42]. The role of CYB-3 in regulating centrosome maturation and differential mitotic entry of maternal and paternal pronuclei is an exciting question that will be addressed in detail in a forthcoming manuscript (Deyter *et al.*, in preparation).

### CYB-3 is required for timely mitotic progression and anaphase onset

We quantified the duration of the first mitotic division in OD57 embryos treated with *control* or *cyb-3(RNAi)* using specific mitotic landmarks as follows: Prophase: interval between pronuclear meeting (PNM; the initial joining of the maternal and paternal pronuclei) and nuclear envelope breakdown (NEB; the absence of clearly demarcated nucleoplasm surrounded by a nuclear envelope); Prometaphase: interval between NEB and chromosome congression to the metaphase plate; Metaphase: interval between complete (or nearly complete) congression and the initiation of anaphase chromosome segregation; Anaphase: interval between the initiation of chromosome segregation and the beginning of chromosome decondensation; Telophase/mitotic exit: interval between the initiation of chromosome decondensation and centrosome breakdown.

**Figure 3:**
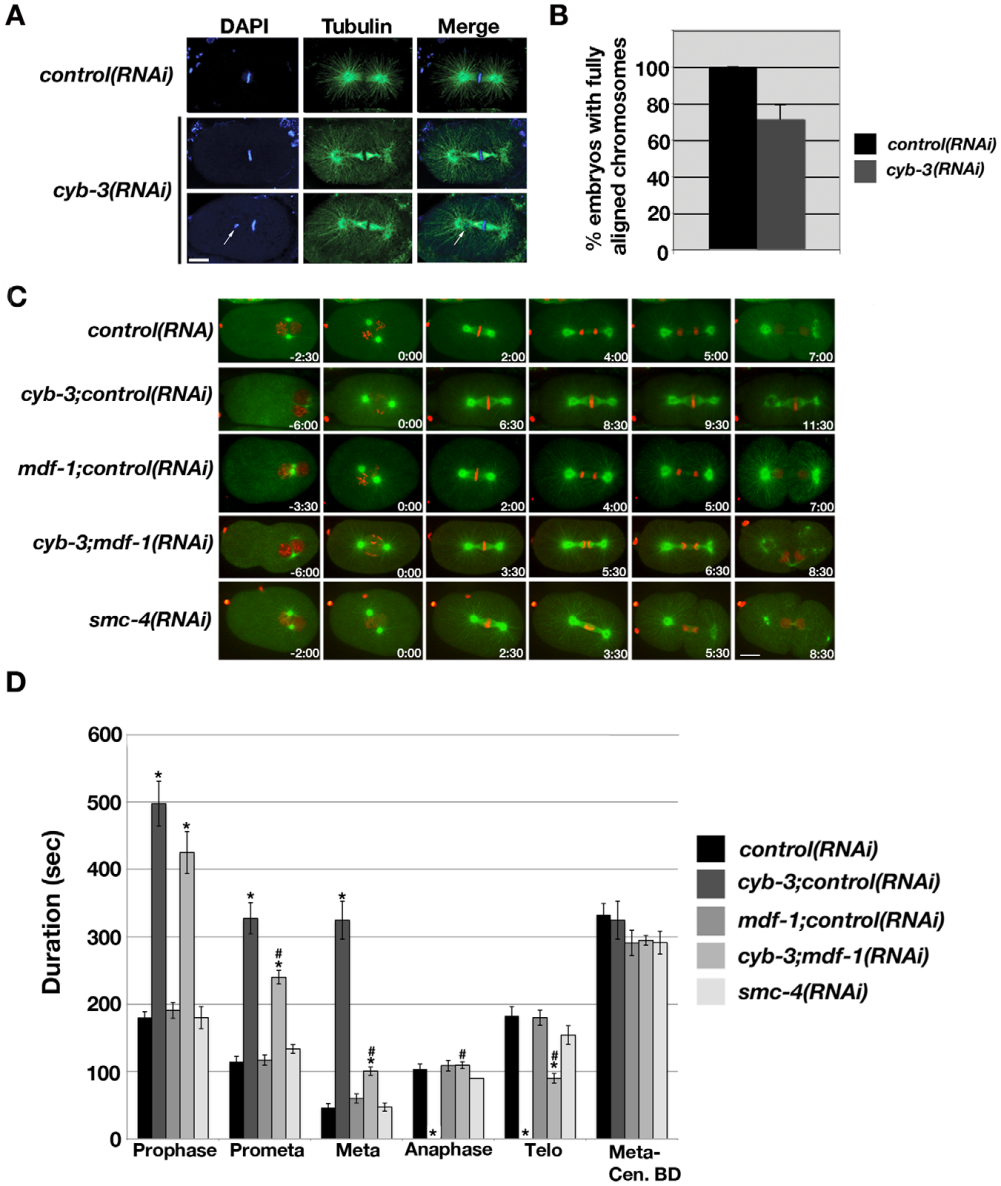
*cyb-3(RNAi)* results in chromosome congression defects and a prolonged SAC–dependent metaphase delay. A) *Control* and *cyb-3(RNAi)* embryos were fixed and stained with DAPI (blue) and an α-tubulin (green) antibody. One-cell embryos at metaphase are shown. Arrow: unaligned chromosome. B) Percentage of fixed *control* and *cyb-3(RNAi)* one-cell embryos with complete metaphase chromosome alignment (*control(RNAi)*, n = 25; *cyb-3(RNAi)*, n* = *42; Error bars: SEM; p = 0.001). C) Selected live images of OD57 embryos treated with the indicated RNAi combinations. 0:00 =  NEB. Images immediately to the left of 0:00 correspond to PNM. D) Duration of mitotic stages after treatment with various RNAi combinations. *: p<0.05 compared to *control(RNAi)*; #: p<0.05 compared to *cyb-3*;*control(RNAi)*. n =  embryos; Error bars: SEM; *control*, n = 7; *cyb-3;control(RNAi)*, n = 8; *mdf-1;control(RNAi)*, n = 10; *cyb-3;mdf-1(RNAi)*, n = 17; *smc-4(RNAi)*, n = 7. Scale bars: 10 µm.

Fixed-cell and live imaging revealed that prophase and prometaphase were approximately two-to-three fold longer in *cyb-3(RNAi)* embryos compared to control, and chromosome congression was often incomplete (Figure 3; Videos S5, S6). 30% of *cyb-3(RNAi)* embryos had at least one chromosome that initially congressed to the metaphase plate but subsequently underwent movement towards the centrosome, followed by re-alignment in the majority of embryos (Figure 3A, 3B; Video S6). *cyb-3(RNAi)* mitotic spindles also had an abnormal appearance, with microtubule bundles appearing to be pinched at the centrosomes rather than the more spread out, straight microtubules of control spindles (Figure 3A). The centrosome-centrosome distance at metaphase was also much greater (Figure 3A and below). However, the most striking and unusual phenotype was the prolonged metaphase delay (Figure 3, Figure S3; Video S6). While metaphase was the shortest mitotic stage in control embryos (Figure 3C, 3D, Figure S3, Video S5), loss of CYB-3 resulted in a prolonged metaphase delay characterized by the persistence of aligned, condensed chromosomes even after other cell cycle events had proceeded (*i.e*., spindle disassembly) (Figure S3, Video S6). The pinched spindle pole phenotype appears to be a function of time spent in metaphase since it becomes more apparent over the course of the delay (Video S6). Since anaphase chromosome segregation and telophase decondensation did not occur, metaphase in *cyb-3(RNAi)* embryos was defined as the continued alignment of chromosomes at the metaphase plate until centrosome breakdown (Figure S3, Video S6). In these embryos, cleavage furrow ingression occurred while chromosomes remained condensed and aligned at the metaphase plate. Indeed, the cleavage furrow often “cut” these chromosomes depending on their position relative to the furrow (Video S6). In addition, centrosomes in *cyb-3(RNAi)* embryos were disassembled only to reform, separate, and nucleate microtubules in the presence of aligned chromosomes (Video S6). These results indicate that the absence of chromosome segregation does not prevent other cell cycle events from proceeding.

To address whether the persistent metaphase delay is a secondary consequence of the failure of the oocyte nucleus to undergo the MII meiotic division, we assayed cell cycle progression in the relatively rare *cyb-3(RNAi)* embryos with two extruded polar bodies, which is indicative of complete MI and MII divisions. All of these embryos (n = 7) displayed metaphase delays comparable to *cyb-3(RNAi)* embryos with single polar bodies (Figure S4). Hence, the failure to undergo mitotic anaphase chromosome segregation does not correlate with increased embryonic ploidy or a failure to undergo the MII division. These results are consistent with the absence of prolonged mitotic metaphase delays in other genetic conditions that disrupt the meiotic divisions of the oocyte nucleus and/or polar body extrusion [43]–[46].

CYB-3 is one of four B-type cyclins in *C. elegans* [30]. The other three Cyclin B proteins include CYB-1, the closest homolog to mammalian B1, and two B2-like proteins. *cyb-1, cyb-2.1*, and *cyb-2.2* are highly similar to one another and were targeted for RNAi elimination via microinjection of a single dsRNA (Figure S1B). As recently described [30], the meiotic divisions were aberrant in *cyb-1&2(RNAi)* embryos (data not shown). However, in sharp contrast to *cyb-3(RNAi)*, *cyb-1&2(RNAi)* mitotic chromosomes did not align to a metaphase plate but still underwent anaphase (Figure S3A; Video S7). Surprisingly, the interval between NEB and the onset of anaphase spindle elongation in CYB-1&2-depleted embryos was similar to controls (*control*: avg.  = 161±26 seconds, number of embryos (n) = 11; *cyb-1&2(RNAi)*: avg.  = 124±20 seconds, n = 7), suggesting that there were no appreciable delays in prometaphase or the metaphase-to-anaphase transition. In conclusion, embryos depleted of CYB-3 exhibit a phenotype distinct from that caused by co-depletion of CYB-1 and CYB-2.

### Anaphase chromosome segregation is restored to *cyb-3(RNAi)* embryos when the spindle assembly checkpoint is compromised

Since the spindle assembly checkpoint (SAC) delays the metaphase-to-anaphase transition in the presence of unattached kinetochores or defective microtubule attachments, we asked whether the prolonged metaphase delay in *cyb-3(RNAi)* embryos was dependent on a functional SAC. Hence, OD57 embryos co-depleted of CeMad1/MDF-1 [47] and CYB-3 were subjected to live imaging (Figure 3C, 3D; Videos S8, S9, S10). As controls, *cyb-3* and *mdf-1* dsRNA-expressing bacteria were diluted with *control* bacteria (see Materials and Methods). Consistent with previous reports, *mdf-1+control(RNAi)* did not result in any apparent defects in the timing or execution of mitosis as compared to *control(RNAi)* embryos (Figure 3C, 3D; Video S9) [48]. The mitotic defects of *cyb-3+control(RNAi)* embryos were indistinguishable from undiluted *cyb-3(RNAi)* (Figure 3C, 3D, and Figure S3; Videos S6, S8). MDF-1 contributed to the prometaphase delay in *cyb-3(RNAi)* embryos, since the duration of prometaphase in *cyb-3+mdf-1(RNAi)* embryos was shortened compared to *cyb-3+control(RNAi)*; however, this interval remained lengthened as compared to *control(RNAi)* (Figure 3C, 3D; Videos S5, S8, S10). Strikingly, *cyb-3+mdf-1(RNAi)* embryos entered anaphase after a brief metaphase delay, suggesting that the SAC is required for the prolonged metaphase in *cyb-3(RNAi)* embryos (Figure 3C, 3D; Videos S8, S10). Indeed, co-depletion of CYB-3 and other SAC proteins (CeMad3/SAN-1 and CeBub1/BUB-1 [49], [50]) also resulted in anaphase onset (Videos S11, S12).

To confirm these results, homozygous *unc-46(e177)*; *mdf-1(gk2)* L4 hermaphrodite offspring (F1) from *unc-46(e177);mdf-1(gk2)* heterozygous mothers were fed *control* or *cyb-3* dsRNA-expressing bacteria. *unc-46(e177)* is a recessive linked visible marker for homozygous *mdf-1(gk2)* animals [47]. *gk2* is a strong loss-of-function deletion allele of *mdf-1* [47]. F1 *mdf-1(gk2)* homozygotes are viable but display a low level of sterility (23%), while the majority of F2 *mdf-1(gk2)* progeny arrest as embryos or larvae [47]. Embryos of RNAi-treated F1 *unc-46(e177)*;*mdf-1(gk2)* or *unc-46(e177)* hermaphrodites were fixed, immunostained with kinetochore (CeBub1/BUB-1)[10] and spindle (α-tubulin) antibodies, and the number of one-cell embryos in mitotic metaphase versus other cell cycle stages was counted (Table 1). 100% of *cyb-3(RNAi);unc-46(e177)* one-cell embryos were in mitotic metaphase and none in anaphase, while 50% of *cyb-3(RNAi);unc-46(e177)mdf-1(gk2)* one-cell embryos were in mitotic metaphase and 36% were in anaphase (Table 1). These data are consistent with the RNAi experiments described above where depletion of MDF-1 results in a significant but not complete reduction in the duration of the extended metaphase in *cyb-3(RNAi)* embryos and permits anaphase onset.

**Table 1:** Number of metaphase and anaphase embryos in CYB-3-depleted *mdf-1(gk2)* embryos.

Genotype	One-cell mitotic embryos		
	n (total number)*	Metaphase	Anaphase
*unc-46(e177);control(RNAi)*	8	1 (12.5%)	2 (25%)
*unc-46(e177);mdf-1(gk2);control(RNAi)*	8	1 (12.5%)	3 (38%)
*unc-46(e177);cyb-3(RNAi)*	20	20 (100%)	0 (0%)
*unc-46(e177);mdf-1(gk2);cyb-3(RNAi)*	14	7 (50%)	5 (36%)

*Remainder of the embryos were in prophase, prometaphase, or telophase.

Given that CYB-3-depleted embryos display chromosome condensation defects (Figure 1D, Figure 2, Figure S2), mitotic progression of embryos depleted of the condensin subunit SMC-4 [51] was assessed to determine whether condensation defects also lead to significant delays in mitotic progression. These experiments revealed that *smc-4(RNAi)* embryos, although highly defective with respect to chromosome condensation, do not display significant delays at any mitotic stage (Figure 3C, 3D; Video S13). These results suggest that the mitotic delay in *cyb-3(RNAi)* embryos is not a secondary consequence of chromosome condensation defects.

### Loss of CYB-3 leads to altered geometry of the metaphase kinetochore

**Figure 4:**
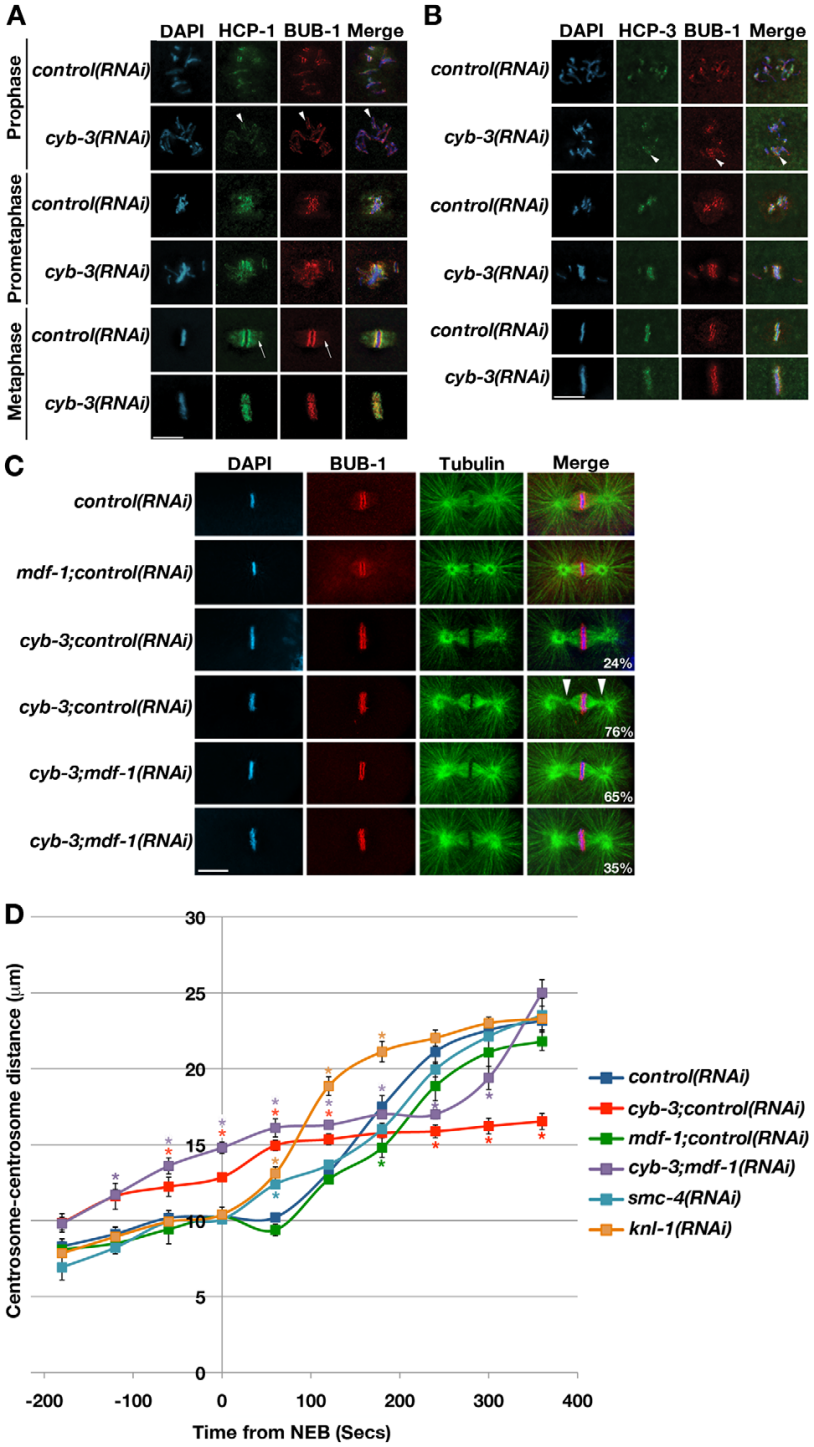
CYB-3 depletion leads to altered metaphase kinetochore geometry and premature spindle pole separation. A) *Control* and *cyb-3(RNAi)* embryos were fixed and stained with DAPI and antibodies recognizing the kinetochore proteins HCP-1 (green) and BUB-1 (red). Arrowheads: resolved sister chromatids (prophase). Arrows: K-Mt immunostaining. B) Embryos treated as in (A) were stained with DAPI and antibodies recognizing HCP-3 (green) and BUB-1 (red). Arrowheads: resolved sister chromatids. C) Embryos treated with the indicated RNAi were fixed and stained with DAPI, and BUB-1 (red) and tubulin (green) antibodies. Arrowheads: pinched spindle poles. Scale bars: 10 µm. D) The centrosome-centrosome distance (µm) in one-cell OD57 embryos treated with the indicated RNAi is plotted with respect to time from NEB (seconds). NEB: 0. Error bars: SEM. *:p<0.05 compared to *control(RNAi)* embryos at the same time-point. n =  embryos. *control(RNAi)*, n = 7; *cyb-3;control(RNAi)*, n = 8; *mdf-1;control(RNAi)*, n = 5; *cyb-3;mdf-1(RNAi)*, n = 7; *smc-4(RNAi)*, n = 7; *knl-1(RNAi)*, n = 4.

*C. elegans* chromosomes are holocentric, providing a large centromere advantageous for studying changes in kinetochore structure and centromere resolution [52]. Given that the organization of kinetochore microtubules was altered in *cyb-3(RNAi)* embryos, we tested whether kinetochore architecture was also changed. Hence, *control* and *cyb-3(RNAi)* embryos were fixed and co-stained with antibodies recognizing two kinetochore proteins, CeCENP-F/HCP-1 [53] and CeBub1/BUB-1 [10] (Figure 4A). In wild-type cells, sister chromatids are resolved from one another in prophase, resulting in paired kinetochores oriented to opposite spindle poles [54]. This geometry lessens the probability of kinetochores interacting with microtubules emanating from the wrong spindle pole. Sister chromatid resolution occurred in both control and CYB-3-depleted embryos, as evidenced by parallel stripes of BUB-1 and HCP-1 staining on prophase chromosomes (Figure 4A, arrowheads). This kinetochore geometry was maintained in both types of embryos through prometaphase. At metaphase, 100% of control embryos had two clearly defined stripes of BUB-1 and HCP-1 staining, as well as kinetochore microtubule (K-Mt) staining (Figure 4A, arrows). However, the majority (>80%) of *cyb-3(RNAi)* embryos had no clear BUB-1 or HCP-1 kinetochore stripes, and no BUB-1 or HCP-1 localization to metaphase K-Mts (Figure 4A). Rather, BUB-1 and HCP-1 staining appeared to be “twisted” and was coincident with the body of the metaphase chromosomes (Figure 4A). Immunostaining with additional kinetochore-specific antibodies (*e.g.*, α-KNL-2) [55], as well as live imaging of GFP::KBP-4^Ndc80^ (strain OD11) transgenic embryos [56], confirmed these results (data not shown).

As in other organisms, the *C. elegans* kinetochore is built on centromeric chromatin containing the histone variant CENP-A (CeHCP-3) [57]. To determine whether the altered metaphase kinetochore architecture in CYB-3-depleted embryos coincided with changes in centromere geometry, fixed embryos were co-stained with BUB-1 and HCP-3 antibodies. These experiments revealed that HCP-3 behaves identically to BUB-1 and HCP-1, suggesting that metaphase kinetochores and underlying centromeres are equally affected by the loss of CYB-3 (Figure 4B). Similar results were obtained upon live imaging of GFP::HCP-3;mCherry::H2B embryos (strain JS9670)[55] (data not shown).

Since the prolonged metaphase delay in *cyb-3(RNAi)* embryos is dependent on the spindle assembly checkpoint, we determined whether depletion of SAC components affected kinetochore geometry. As above, the majority of one-cell embryos treated with *cyb-3+control(RNAi)* displayed “twisted” kinetochores (Figure 4C). The twisting appears to increase over the course of the delay since it correlates with the severity of the pinched spindle pole phenotype (compare the two *cyb-3;control(RNAi)* embryos in Figure 4C). Co-depletion of CeMad1/MDF-1 resulted in a suppression of both phenotypes, with 65% of co-depleted one-cell embryos displaying two distinct stripes of BUB-1 staining and normal spindle morphology (Figure 4C). Note that while BUB-1 was localized to metaphase K-Mts in *control* and *mdf-1+control(RNAi)* embryos, no K-Mt BUB-1 localization was apparent in *cyb-3(RNAi)* or *cyb-3+mdf-1(RNAi)* embryos.

The quality of kinetochore-microtubule attachments in *C. elegans* is directly reflected by the timing and rate of spindle pole separation. *C. elegans* chromosomes do not undergo anaphase A movements [35]; therefore, cortical pulling forces on centrosomes and astral microtubules prior to anaphase are countered by bipolar kinetochore-microtubule attachments to cohesed sister chromosomes. When kinetochore-microtubule attachments are defective, spindle poles separate immediately upon NEB as there are no forces counteracting astral microtubule-based pulling of centrosomes to the cell cortex [35]. For instance, in embryos depleted of the core kinetochore protein KNL-1, the distance between centrosomes rapidly increases immediately after NEB (Figure 4D and [58]). Interestingly, the centrosome-centrosome distance in *cyb-3(RNAi)* embryos was significantly greater than control embryos prior to NEB (Figure 4D), indicating that CYB-3 is likely to be affecting processes other than or in addition to kinetochore-microtubule interactions (see below). Indeed, this premature spindle pole separation was not affected by abrogation of the spindle assembly checkpoint (Figure 4D). Spindle length in *cyb-3(RNAi)* embryos is stabilized within 60 seconds after NEB at the same length (14.5 µm±0.73 (SEM); n = 6) as the metaphase-to-anaphase transition spindle in control embryos (15.7 µm±0.56 (SEM); n = 6; p = 0.2)(180 seconds post-NEB)(Figure 4D). Spindles in embryos co-depleted of CYB-3+MDF-1 behaved similarly to *cyb-3(RNAi)* spindles until the centrosomes of the former separated coincident with anaphase chromosome segregation (approximately 240 seconds post-NEB) (Figure 4D). Mitotic spindles in SMC-4-depleted embryos undergo a brief premature spindle pole separation just after NEB, but then the centrosome-centrosome distance increases at the same rate as spindles in control embryos (Figure 4D). Hence, the premature spindle pole separation phenotype of CYB-3-depleted embryos is not likely to be a consequence of chromosome condensation defects. Altogether, these data indicate that loss of CYB-3 results in very early, pre-NEB centrosome separation, perhaps due to abnormalities in the attachment of centrosomes to the nuclear envelope (see Discussion). *cyb-3(RNAi)* spindles then stabilize at the same length as control metaphase spindles (180 seconds post-NEB), indicating that kinetochore-microtubule interactions reach levels that balance cortical pulling forces similarly to control spindles. This balance could also be achieved if kinetochore-microtubule interactions were compromised coincident with a diminution of cortical pulling forces. However, spindle pole separation and sister chromatid segregation in *cyb-3+mdf-1(RNAi)* embryos are not consistent with this latter model. While we cannot rule out the presence of underlying spindle abnormalities or assembly defects, these data reveal that CYB-3-depleted embryos are capable of generating at least grossly functional kinetochore-microtubule attachments.

### Metaphase chromosomes in *cyb-3(RNAi)* embryos accumulate spindle checkpoint proteins and dynein

**Figure 5:**
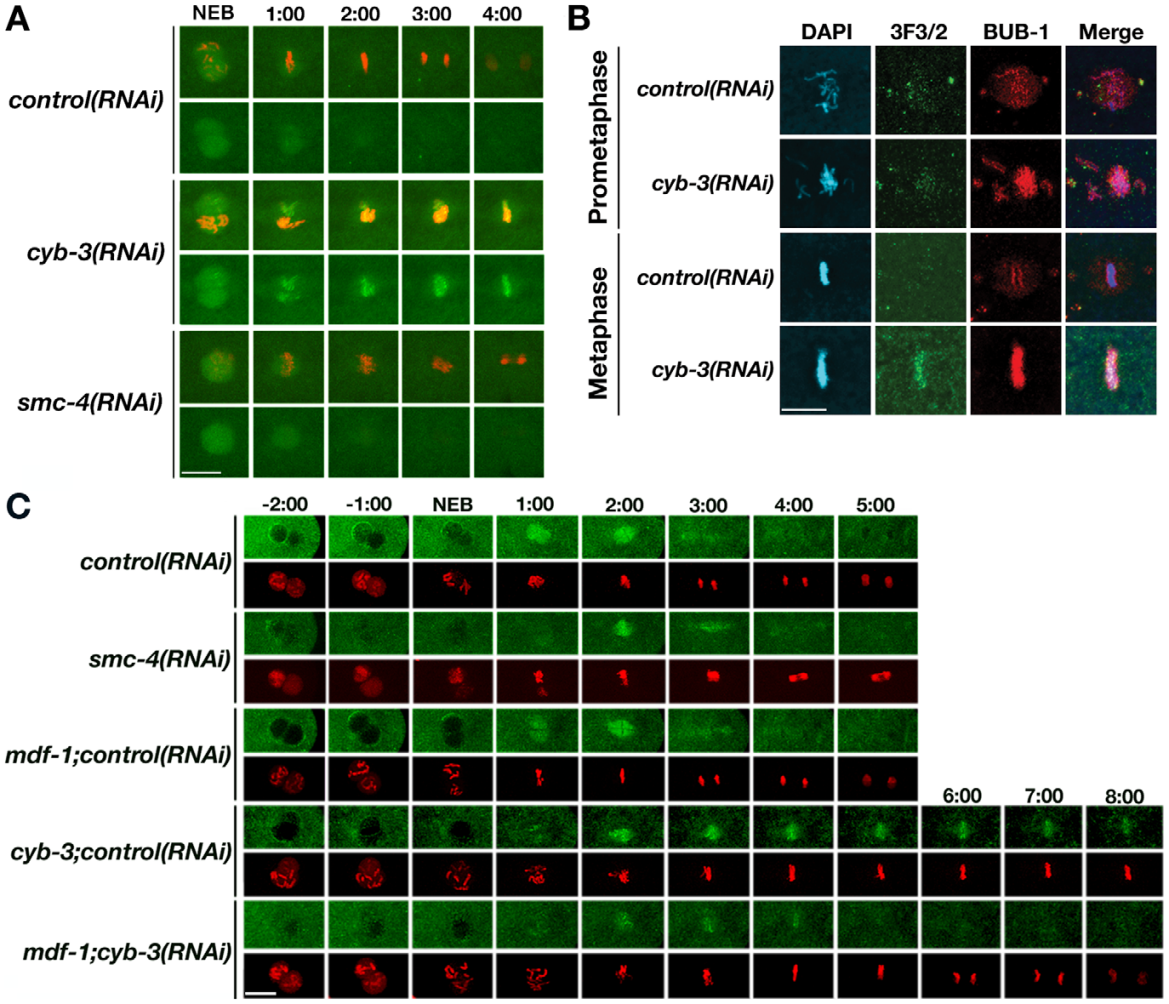
SAC proteins and dynein accumulate at kinetochores in *cyb-3(RNAi)* embryos. A) GFP::MDF-2;mCherry::H2B (OD110) embryos treated with the indicated RNAi were subjected to live imaging. Top panel for each RNAi condition: GFP::MDF-2+mCherry::H2B; bottom panel: GFP::MDF-2 alone. 0:00: NEB, all other images are one minute intervals post-NEB. B) *Control* and *cyb-3(RNAi)* embryos were fixed and stained with DAPI (blue), and 3F3/2 (green) and BUB-1 (red) specific antibodies. C) Selected images from videos of GFP::DHC-1;mCherry::H2B)(OD203) embryos treated with the indicated RNAi. Top panel for each RNAi condition: GFP::DHC-1; bottom panel: mCherry::H2B. 0:00: NEB. Scale bars: 10 µm.

The spindle assembly checkpoint delays anaphase entry until all chromosomes achieve bipolar attachment to the mitotic spindle [4]. In mammalian cells, this delay can be several hours [9]. However, *C. elegans* SAC-dependent mitotic delays are transient. The worm SAC mediates a modest two-fold increase in the interval between NEB and anaphase onset, even when kinetochore-microtubule attachments are severely compromised by treatment with the microtubule inhibitor nocodazole [50]. The complete SAC-dependent abrogation of anaphase spindle elongation and chromosome segregation in CYB-3-depleted cells suggests that loss of CYB-3 results in a much “stronger” and/or persistent checkpoint response. Since the checkpoint protein CeMad2/MFD-2 is recruited to unattached chromosomes and is “stripped” from kinetochores upon microtubule attachment and checkpoint satisfaction [9], [10], [20], we examined the localization of GFP::MDF-2 in living *C. elegans* embryos (strain OD110) treated with *control* or *cyb-3(RNAi).* As previously reported, GFP::MDF-2 localizes to prophase and prometaphase nuclei but is not apparent on metaphase kinetochores in control embryos (Figure 5A; Videos S14, S15 and [10]). Interestingly, in *cyb-3(RNAi)* embryos, GFP::MDF-2 accumulated on chromosomes beginning in prophase and remained on chromosomes throughout the prolonged metaphase in these cells (Figure 5A; Videos S16, S17). In SMC-4-depleted embryos, GFP::MDF-2 behaved similarly to control cells, indicating that reduced chromosome condensation does not lead to the retention of MDF-2 on metaphase chromosomes (Figure 5A: Videos S18, S19).

A second hallmark of an engaged SAC is the accumulation of phospho-specific epitopes recognized by the 3F3/2 antibody [11], [59], [60], which is thought to correlate with reduced tension within and across paired sister kinetochores [8], [61]. As expected, 3F3/2 immunostaining of chromosomes increased upon taxol treatment (Figure S5), indicating that this antibody recognizes epitopes in *C. elegans* that are sensitive to microtubule dynamics. 3F3/2 immunostaining accumulated around prophase and prometaphase chromosomes in *control* and *cyb-3(RNAi)* treated embryos (Figure 5B and data not shown). While staining was absent in control cells at metaphase, it accumulated to high levels on metaphase chromosomes in *cyb-3(RNAi)* embryos, consistent with persistent SAC signaling.

The ability of checkpoints to halt cell cycle progression in response to DNA damage and spindle assembly defects is well established [7], [62]. In the past few years, it has become apparent that cells must not only satisfy these checkpoints (*e.g.*, attach all chromosomes) but also actively silence these checkpoints once the damage or defects have been repaired [18], [63], [64]. The minus-end directed microtubule motor dynein contributes to SAC inactivation by trafficking SAC components from kinetochores along K-Mts to centrosomes [20], [23]. Since SAC proteins accumulate on metaphase chromosomes in CYB-3-depleted cells, we wondered whether dynein was appropriately localized in *cyb-3(RNAi)* embryos; hence, we examined dynein behavior in *C. elegans* embryos harboring a GFP-tagged dynein heavy chain (GFP::DHC-1) transgene (strain OD203)[65]. In control embryos, GFP::DHC-1 localized to the nuclear periphery in prophase and was associated with chromosomes upon nuclear envelope breakdown (Figure 5C; Videos S20, S21). At metaphase, kinetochore and K-Mt localization was evident. At anaphase, GFP::DHC-1 was no longer detectable at kinetochores but was still apparent on K-Mts and centrosomes (Figure 5C, Videos S20, S21). A similar pattern was found in embryos treated with *smc-4(RNAi)* or *mdf-1+control(RNAi)* (Figure 5C, Videos S22, S23, S24, S25). In *cyb-3(RNAi)* embryos, GFP::DHC-1 localized to the nuclear periphery and centrosomes during prophase and also accumulated at kinetochores during prometaphase and metaphase as in controls (Figure 5C, Videos S26, S27). Strikingly, there was little or no apparent localization to K-Mts or centrosomes at anytime after NEB. In contrast, GFP::DHC-1 was readily apparent on kinetochores in embryos co-depleted of MDF-1+CYB-3 but disappeared just prior to anaphase initiation; no localization to K-Mts or centrosomes was apparent (Figure 5C, Videos S28, S29). Immunostaining with an antibody specific for Dynactin/DNC-1 led to similar results (Figure S6).

The inability of dynein and dynein-related proteins to associate with the mitotic spindle in *cyb-3(RNAi)* embryos could reflect a global defect in microtubule-associated proteins (MAPs) binding to K-Mts. However, the CeBimC/BMK-1 kinesin [66] localizes to K-Mts in both *control* and *cyb-3(RNAi)* embryos (Figure S7), indicating that K-Mts in CYB-3-depleted embryos are not inaccessible to microtubule motors.

Altogether, these data are consistent with a model whereby loss of CYB-3 leads to persistent chromosomal SAC signaling, characterized by a failure of dynein and SAC proteins to mobilize from kinetochores to K-Mts and centrosomes, leading to a robust block in anaphase chromosome segregation.

### Modulation of dynein activity coordinately affects cell cycle progression in CYB-3–depleted embryos

**Figure 6:**
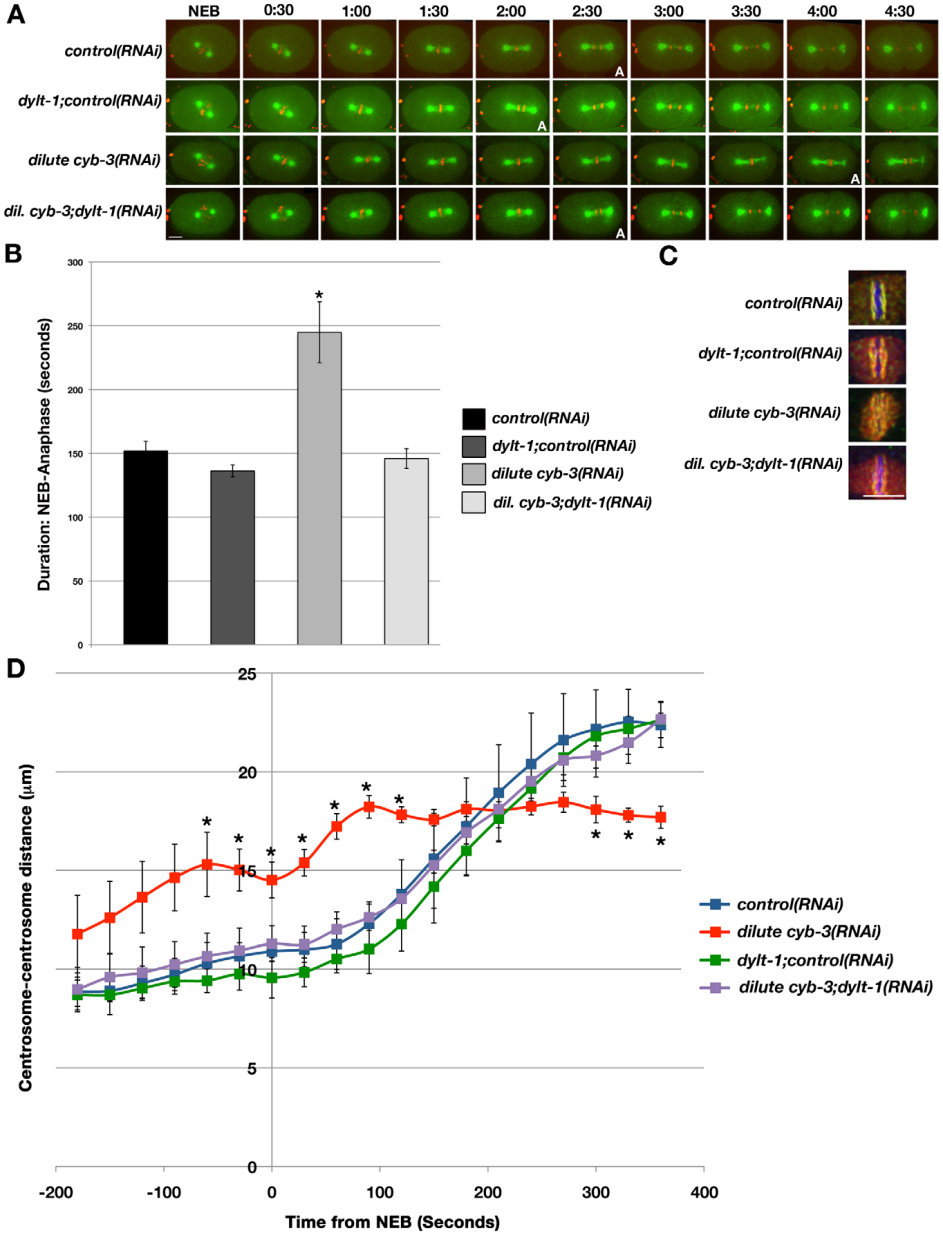
Modulation of dynein activity alters cell cycle progression and the rate of spindle pole separation in CYB-3–depleted embryos. A) Selected live images of OD57 embryos treated with the indicated RNAi. 0:00: NEB. A: anaphase entry. Scale Bar: 10 µm. B) Time from NEB-anaphase entry (seconds) in OD57 embryos treated with the indicated RNAi. Error bars: SEM; n =  embryos; *control(RNAi)*, n = 11; *dylt-1;control(RNAi)*, n = 11; *dilute cyb-3(RNAi)*, n = 6; *dilute cyb-3;dylt-1(RNAi)*, n = 7; Scale bar: 10 µm. *: p<0.05 as compared to control embryos. C) Embryos treated with the indicated RNAi conditions were fixed and stained with DAPI (blue), and BUB-1 (red) and HCP-1 (green) antibodies. n =  number of twisted metaphase plates/number of embryos examined. *control(RNAi)*, n = 0/5; *dylt-1+control(RNAi)*, n = 1/5; *dilute cyb-3(RNAi)* n = 5/5; *dilute cyb-3+dylt-1*, n = 0/3. Scale bar: 5 µm. D) The centrosome-centrosome distance (µm) in one-cell OD57 embryos treated with the indicated RNAi is plotted with respect to time from NEB (seconds). NEB: 0. Error bars: SEM. *:p<0.05 compared to *control(RNAi)* embryos at the same time-point. n =  embryos. *control(RNAi)*, n = 8; *dylt-1;control(RNAi)*, n = 5; *dilute cyb-3(RNAi)*, n = 7; *dilute cyb-3;dylt-1(RNAi)*, n = 6.

One possible mechanistic model that explains our findings is that CYB-3 directly or indirectly promotes dynein activity with respect to SAC satisfaction and/or silencing. One prediction of this model is that increasing dynein activity should alleviate SAC signaling in *cyb-3(RNAi)* embryos, leading to timely anaphase entry. A recent study in *C. elegans* revealed that specific dynein light chains negatively regulate the activity of the dynein heavy chain (DHC-1) [67]. Although loss of the light chain DYLT-1 leads to no discernible phenotype in a wild-type background, DYLT-1 depletion rescues the lethality of a temperature-sensitive (ts) *dhc-1* allele [67]. Hence, we determined whether co-depleting DYLT-1 with CYB-3 would affect the ability of these cells to enter anaphase. To increase the sensitivity of our assay, we diluted *cyb-3* dsRNA bacteria 20-fold with *control* or *control*+*dylt-1* dsRNA bacteria and fed these mixtures to young adult OD57 hermaphrodites. Embryos treated with diluted *cyb-3(RNAi)*(20x dilution with *control* bacteria) experienced significant metaphase delays, but ultimately underwent anaphase chromosome segregation approximately 90 seconds after control embryos (Figure 6A, 6B; Video S30). However, diluting *cyb-3* dsRNA bacteria 20x with *control+dylt-1* dsRNA bacteria completely abrogated this delay, leading to anaphase onset coincident with controls (180 seconds post-NEB; Videos S31, S32). Furthermore, while kinetochore twisting was readily apparent in embryos treated with dilute *cyb-3(RNAi)*, this phenotype was rescued by concomitant loss of DYLT-1 (Figure 6C).

As described above, the distance between mitotic centrosomes in CYB-3-deficient embryos begins to increase well before NEB, and this distance remains significantly greater than in wild-type embryos until stabilizing with the same spacing as centrosomes of wild-type spindles at the metaphase-to-anaphase transition (Figure 4D and Figure 6D). Interestingly, loss of DYLT-1 rescued the premature centrosome separation phenotype of embryos treated with dilute *cyb-3(RNAi)*, both before and after NEB (Figure 6D). Altogether, the rescue of these abnormalities and abrogation of the metaphase delay by modulating dynein functionality reveal that *cyb-3* genetically interacts with components of the dynein motor complex and support a model whereby CYB-3 promotes the functionality of mitotic dynein with respect to spindle assembly and mitotic progression.

**Figure 7:**
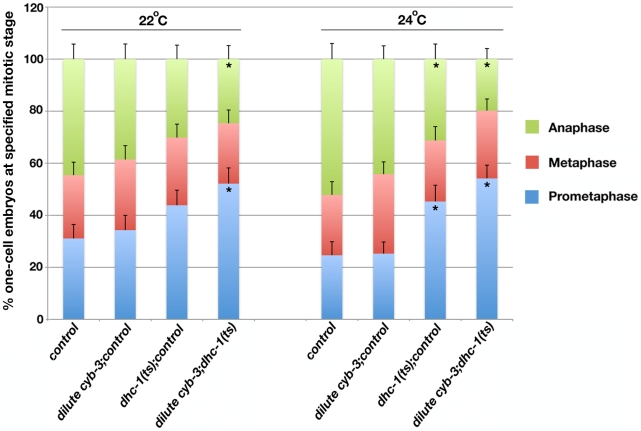
Inhibition of DHC-1 enhances cell cycle progression delays in *cyb-3(RNAi)* embryos. Embryos from wild-type and *dhc-1(ts)* hermaphrodites treated with the indicated RNAi were fixed and stained with DAPI and an α-tubulin antibody. % one-cell embryos at prometaphase, metaphase, and anaphase (reared at 22°C or 24°C). n =  total number of one-cell embryos; *control* (wild-type + *control(RNAi)*), n = 74 (22°C), 69 (24°C); dilute *cyb-3*;*control* (wild-type+*dilute cyb-3(RNAi)*), n = 70 (22°C), 95 (24°C); *dhc-1(ts)*;*control* (*dhc-1(ts)*+*control(RNAi)*), n = 73 (22°C), 64 (24°C); diluted *cyb-3;dhc-1(ts)* (*dhc-1(ts)* + *dilute cyb-3(RNAi)*), n = 69 (22°C), 96 (24°C). Error bars: SEM. *: p<0.05 as compared to control embryos at the same temperature.

If *cyb-3* genetically promotes dynein activity, then we predict that dynein impairment would enhance *cyb-3(RNAi)* phenotypes. Hence we utilized a *dhc-1(ts)* allele to test this model. Embryos from *dhc-1(ts)* hermaphrodites reared at semi-permissive temperatures (22°C and 24°C) were fed *cyb-3* dsRNA bacteria diluted 20x with *control* bacteria. Embryos were fixed after 24 hours on dsRNA bacteria and the number of one-cell embryos at different stages of mitosis was counted (Figure 7). With respect to embryos reared at 22°C, there were no statistically significant differences in the number of one-cell embryos in prometaphase, metaphase, or anaphase between wild-type or *dhc-1(ts)* embryos treated with *control(RNAi)* or wild-type embryos treated with diluted *cyb-3(RNAi)*. However, in *dhc-1(ts)* embryos treated with diluted *cyb-3(RNAi)*, there was a significant increase in the number of prometaphase embryos and a concomitant decrease in the number of anaphase embryos (Figure 7). Embryos reared at 24°C revealed similar distributions with the exception that *dhc-1(ts)* embryos treated with *control(RNAi)* also showed a significant increase in the number of prometaphase embryos and a decrease in anaphase embryos (Figure 7). Since DHC-1 inhibition slows the rate of prometaphase (Figure 7 and [22]), the increase in the number of prometaphase embryos from animals co-depleted of CYB-3 and DHC-1 is satisfyingly consistent with a model whereby CYB-3 plays a critical, positive role in the regulation of dynein during mitosis.

## Discussion

Here, we report that *C. elegans* CYB-3 plays an essential role in the timing and execution of many mitotic events in the early embryo, including pronuclear migration, chromosome condensation, centrosome maturation, spindle pole separation, chromosome congression, and alleviation of a SAC-dependent block in the initiation of anaphase chromosome segregation. In addition, genetic experiments are consistent with *cyb-3* acting as a direct or indirect positive regulator of mitotic dynein functionality.

Given that other cyclins have a variety of targets, it is not surprising that CYB-3 affects a number of different cellular events. A commonality of many of these processes is that they are dynein-dependent. The significantly slower migration rate of the female pronucleus and the failure of the male pronucleus to move from the embryo posterior in CYB-3-depleted embryos are strikingly similar to the pronuclear defects of *C. elegans* embryos harboring a temperature-sensitive allele of the dynein heavy chain *dhc-1* [22]. Moreover, centrosome attachment to the nuclear envelope is also dynein-dependent [68]–[70]. The pre-NEB increase in centrosome-centrosome distance in *cyb-3(RNAi)* embryos and rescue of this phenotype by modulation of dynein activity suggest that centrosome attachment to the nuclear envelope is compromised in CYB-3-deficient embryos. Consequently, the shortening of the post-NEB centrosome-centrosome distance in *cyb-3;dylt-1(RNAi)* compared to *cyb-3(RNAi)* embryos may be a secondary consequence of stronger centrosome-nuclear envelope attachments and thus abrogation of pre-NEB separation. Alternatively, it may reflect the more timely formation of, or more robust, kinetochore-microtubule attachments than in CYB-3-depleted embryos. Unfortunately, our data neither allow us to distinguish between these two possibilities nor address whether modulating dynein activity also affected CYB-3-dependent prophase events since diluted *cyb-3(RNAi)* embryos did not display consistent defects in early mitosis. Experiments to titrate the amount of CYB-3 and dynein activity required for different mitotic events are underway.

Although loss of CYB-3 affects a number of processes, the most striking abnormality is the persistent SAC-dependent delay in the initiation of anaphase chromosome segregation. To our knowledge, this is an unprecedented phenotype in the early *C. elegans* embryo. Several groups have reported that the *C. elegans* SAC is relatively weak and can only mount, at most, a two-to-three fold delay in the metaphase-to-anaphase transition under all conditions tested, including severe spindle damage after nocodazole exposure [10], [47], [48], [50]. Two potential mechanisms to explain the unusual duration of the SAC-dependent metaphase delay in CYB-3-depleted embryos are: 1) loss of CYB-3 results in rare or very specific spindle defects that engage the SAC more persistently than other mitotic spindle abnormalities reported to date, or 2) CYB-3 is required for SAC inactivation or silencing.

Loss of CYB-3 leads to gross defects in kinetochore and kinetochore-microtubule architecture. We posit that the twisted centromeres and kinetochores are the result of multiple cycles of microtubule attachment and detachment occurring during the metaphase delay. Microtubule attachment appears to play a role since prophase kinetochores are not affected by CYB-3 depletion and the twisting correlates with increased AIR-2/Aurora B activity (G.M.R.D, unpublished), which is congruent with increased kinetochore-microtubule turnover. This twisting is phenotypically distinct from that seen in embryos depleted of the condensin proteins SMC-4 and HCP-6 [71] (G.M.R.D, unpublished) and is consistent with findings that centromeres and kinetochores are not elastic [72]. These twisted kinetochores could potentially lead to unusually persistent SAC signaling and a prolonged metaphase delay. However, our data suggest that kinetochore-microtubule attachments are stabilized or reach a steady state since the centrosome-centrosome distance in *cyb-3(RNAi)* embryos reaches and is maintained at the same length as control spindles at the metaphase-to-anaphase transition. Furthermore, when the metaphase delay is abrogated by loss of the SAC or DYLT-1, twisting is not apparent and sister chromatid separation readily occurs, suggesting that kinetochore-microtubule attachments are made and are at least partially functional. Hence, although CYB-3 loss may very well lead to spindle assembly defects that engage the SAC, the unusual persistence of SAC signaling in this circumstance and not in embryos with qualitatively more severe spindle and chromosome segregation defects is not easily reconciled.

A second potential mechanism, and one we favor, is that among other essential mitotic functions, CYB-3 is required to inactivate or silence the SAC. Our data are consistent with a model whereby CYB-3 participates in SAC silencing by either directly or indirectly affecting the ability of dynein to strip SAC components from kinetochores. The dynein motor has been implicated in SAC silencing in mammals, *C. elegans*, and *Drosophila* [20]–[22], [73]. Dynein, dynein-regulatory proteins, and SAC components all accumulate on metaphase kinetochores but do not appear to transfer to K-Mts or to centrosomes in CYB-3-depleted embryos, consistent with a conserved role for dynein in SAC silencing [20]–[22], [73]. Suppression of the metaphase delay by depleting a dynein inhibitor supports a working model that CYB-3 is a positive regulator of dynein *vis-à-vis* SAC silencing. Furthermore, a role for CYB-3 in SAC silencing is more easily reconciled with the rare and unusually persistent metaphase delay in CYB-3-depleted embryos. While a plethora of proteins are required for spindle assembly [74], relatively few have been shown to be required for SAC silencing [18], [20], [21], [75], [76]. Hence, while loss of many different proteins leads to spindle defects and transient engagement of the SAC in the *C. elegans* embryo, many fewer would be necessary to turn off the SAC and allow cell cycle progression. It will be very interesting to determine whether proteins implicated in SAC silencing in other systems, such as the phosphatase PP1 [75], [77], also lead to prolonged SAC-dependent metaphase delays in the *C. elegans* embryo.

One puzzling aspect of the *cyb-3* loss-of-function phenotype is that despite a complete inhibition of sister chromatid separation and chromosome decondensation, centrosomes breakdown and duplicate with the same timing as mitotic centrosomes in wild-type cells (*i.e.*, at the same time relative to NEB). Engagement of the SAC should inhibit all aspects of cell cycle progression. Strikingly, an uncoupling of the nuclear and centrosome cell cycle occurred upon depletion of *Drosophila* mitotic cyclins [28], [78]. While mitotic entry was inhibited, centrosomes continued to duplicate with the same timing as the wild-type cell cycle. Centrosome duplication even occurred in the presence of an inhibitor of the anaphase promoting complex (APC). In the presence of mitotic cyclins, the same inhibitor led to a block in both the nuclear and centrosome cell cycle [78]. These results suggest that loss of mitotic cyclins eliminates the dependence of the centrosome cycle on an active APC, which is consistent with our findings that centrosome breakdown and duplication continues in the absence of CYB-3 despite an engaged SAC. Recent reports further support a model whereby cyclins and cyclin-dependent kinases “entrain” other cell cycle events and the dependency of these events is disrupted when cyclin or CDK activity is compromised [79], [80].

Altogether, our data demonstrate that CYB-3 plays a distinct, non-redundant role in mitosis by influencing dynein-dependent mitotic processes. That CYB-3 depletion does not mirror all dynein/DHC-1 loss-of-function phenotypes may reflect a requirement for CYB-3 in some dynein-related processes but not others, or may indicate that different processes require varying doses of dynein activity. This hypothesis is supported by the isolation of hypomorphic dynein alleles that display a range and severity of defects [22], [68], [81]. The simplest of multiple possible mechanistic relationships between CYB-3 and dynein would be the direct phosphorylation of dynein subunits by a CYB-3/CDK-1 holoenzyme. In mammalian cells, cyclin B3 associates with both Cdk1 and Cdk2 [24], but a second report suggests that human cyclin B3 binds exclusively with Cdk2; however, this association does not result in detectable kinase activity [25]. *C. elegans* CYB-3 associates with CDK-1 *in vitro*, and CYB-3 complexes display H1 kinase activity; H1 is commonly used as a Cdk1 substrate [30], [33]. Interestingly, a recent study revealed that direct phosphorylation of the human dynein light intermediate chain (LIC1) by Cdk1 activates dynein and promotes Mad2 removal from the kinetochore, leading to SAC inactivation and anaphase progression [23]. Cdk1 complexes isolated from cell extracts phosphorylated LIC1, and while the authors did not identify the specific cyclin cofactor, our results suggest that this phosphorylation may be specifically due to a Cdk1/Cyclin B3 complex. However, of the four Cdk1 phosphorylation sites in LIC [23], only one is partially conserved in the *C. elegans* ortholog, DLI-1. Furthermore, unlike CYB-3 and DHC-1, DLI-1 does not appear to be required for the MII division of the oocyte nucleus [82], suggesting that if DLI-1 is a direct CYB-3/CDK-1 target, then there are certain to be additional substrates. Biochemical studies to address which, if any, of the 13 dynein subunits in *C. elegans* are directly phosphorylated by CYB-3/CDK-1 and the functional consequence of these phosphorylation events on mitotic progression are important investigations for the future.

## Materials and Methods

### *C. elegans* strains

*C. elegans* strains were maintained at 15°C–25°C [83]. The following strains were used: N2 (*C. elegans* wild type, DR subclone of CB original (Tc1 pattern I)) [83], OD57 (*unc-119(ed3); ltIs37 [pAA64: pie-1p::mCherry::his-58+ unc-119 (+)]; ltIs25 [pAZ132; pie-1p::GFP::tba-2+ unc-119 (+)]*) [36], [84], CB177 (*unc-46(e177)* V) [83], KR3627 (*unc-46(e177)mdf-1(gk2)* V/*nT1[let-X]* IV;V) [85], OD110 (*unc-119(ed3) III; ltIs52* [*pOD379; pie-1/GFP::Y69A2AR.30; unc-119 (*+*)*]; *ltIs37* [*pAA64;pie-1/mCherry::his-58; unc-119 (+)*] IV)[10], OD203 (*unc-119(ed3) III; orls17 [dhc-1::GFP::dhc-1; unc-119(+)]; ltIs37 [pAA64; pie- 1/mCherry::his-58; unc-119 (+)] IV*)[65], [86], OD11 (*unc-119(ed3) III; ltIs7 [pIC41; pie-1/GFP-TEV-STag::kbp-4; unc-119(+)]/+*)[65], TH32 (*unc-119(ed3) III; ruIs32 [pAZ132; pie-1/GFP::his-58; unc-119(+)] III; ddIs6 [pie-1/GFP::tbg-1; unc-119(+)]V*) [87], EU828 (*dhc-1*(*or195*) I) [22]. To create the GFP::HCP-3; mCherry::Histone H2B strain (JS967), OD101 [55] and OD56 [10] strains were crossed and animals homozygous for the *pie-1/GFP::hcp-3* and *pie-1/mCherry::his-58* transgenes were isolated.

### RNAi–mediated interference (RNAi)

RNAi plasmids for *cyb-3*, *mdf-1*, *san-1*, *bub-1*, *smc-4*, *knl-1*, and *dylt-1* were obtained from the Geneservice Ltd. *C. elegans* feeding library [88]. The L4440 RNAi vector was used as an RNAi control (*control*). To deplete CYB-3 alone, a three ml LB + 100 µg/µl ampicillin liquid culture was seeded with a single colony of HT115 bacteria transformed with the *cyb-3(RNAi)* L4440 plasmid and shaken overnight (O/N) at 37°C. The next day, the O/N culture was expanded to 50 ml with the same media and grown until the OD600 of the culture was between 0.6–0.8 (∼ two hours). IPTG was added to a final concentration of 1 mM and the culture was grown an additional three hours at 37°C to induce *cyb-3* dsRNA expression. The culture was then centrifuged at 5000 rpm for 10 minutes, the pellet was resuspended in 800 µl LB, and 200 µl of the suspension plated on nematode growth (NG) media containing 100 µg/µl ampicillin and three mM ITPG (NG/AMP/IPTG). Plates were incubated at 37°C O/N and then seeded with L4 larvae. Seeded plates were incubated at 25°C O/N and embryos from the young adult worms (L4+24 hours) were utilized for experiments.

To co-deplete CYB-3 and MDF-1, SAN-1, or BUB-1, the induction conditions were as described above. However, after resuspension of the pellets in 800 µl LB, 200 µl of each suspension (*i.e.*, *cyb-3* and *mdf-1* dsRNA-expressing bacteria) were thoroughly mixed and transferred to NG/AMP/IPTG plates, incubated at 37°C O/N, and then seeded with L4 larvae. To generate highly dilute *cyb-3(RNAi)* conditions for *dylt-1* and *dhc-1(ts)* experiments, *control* and *cyb-3* dsRNA expressing bacteria were induced, pelleted, and resuspended as above. 10 µl *cyb-3(RNAi)* bacteria were thoroughly mixed with 190 µl *control* or *dylt-1(RNAi)* bacteria in a 15 ml conical tube and briefly centrifuged at low speed. The pellet was resuspended in the supernatant and plated as above.

For *cyb-1&2(RNAi)* experiments, sense and anti-sense mRNAs corresponding to ZC168.4 (CYB-1) were transcribed from linearized templates using a T7 *in vitro* transcription kit (Ambion, Austin, TX). Complementary RNAs were mixed, heated at 90°C for five minutes, and annealed at room temperature (RT). *cyb-3* dsRNA was also generated in this manner for direct comparison of injected animals. dsRNAs were injected into the gonads of OD57 L4 larvae and the injected animals incubated at 25°C O/N.

### Immunostaining

Embryos from adult hermaphrodites were fixed and stained as previously described [89]. Primary antibodies used were α-tubulin (Sigma, St. Louis, MO), HCP-1 [53], BUB-1 [90], HCP-3 [90], 3F3/2 (Boston Biologicals, Boston, MA)[60], DNC-1 [91], and BMK-1 [66]. Secondary antibodies were: Alexa Fluor® 488 goat anti-mouse IgG or IgM, and Alexa Fluor® 555 goat anti-rabbit IgG (both at 1∶1000) (Invitrogen Molecular Probes, Eugene, OR). For HCP-3 and BUB-1 co-staining experiments, HCP-3 and BUB-1 antibodies were directly conjugated to fluorophores utilizing the Zenon Tricolor Rabbit IgG labeling kit (Invitrogen Molecular Probes, Eugene, OR) as per the manufacturer's instructions. The labeled antibodies were incubated on slides with fixed embryos for three hours at RT. Slides were washed three times with PBSTb (PBS, 0.1% TritonX-100, 0.1% BSA) and mounted with ProLong Gold with DAPI (Invitrogen Molecular Probes, Eugene, OR).

### Image analysis and live imaging

Immunofluorescent images were acquired on a Nikon 2000U inverted microscope equipped with a Photometrics Coolsnap HQ camera. Metamorph software was used for image acquisition. Z-sections were acquired at 0.2 µm steps using a 60X/1.49 NA objective. Z-stacks were projected and deconvolved for 10 iterations using Autodeblur (Autoquant Media Cybernetics, Bethesda, MD). Images were processed for figures using Adobe Photoshop.

For live imaging, embryos cut from RNAi-treated hermaphrodites (24 hours post-RNAi exposure) were mounted on 2% agarose pads and imaged using a spinning disk confocal (Perkin Elmer, Waltham, MA) attached to a Nikon TE2000U inverted microscope. Images were acquired using an ORCA-ER digital camera (Hamamatsu, Bridgewater, NJ) and a 60×1.45 NA Plan Apo VC lens. Ultraview software (Perkin Elmer) was used to control the confocal, microscope, and camera. Images were captured at 30 second intervals; Z-sections were 1 µm. For condensation assays, condensation of male pronucleui in TH32 RNAi-treated embryos were imaged and the condensation parameter calculated as previously described [87]. Image J software (http://rsbweb.nih.gov/ij) was used to measure centrosome size, centrosome-centrosome distance, and pronuclear migration rates.

## Supporting Information

Figure S1 *cyb-3(RNAi)* efficiency and an alignment of *C. elegans* B-type cyclins. A) Protein extracts from *control* and *cyb-3(RNAi)* embryos were immunoprecipitated with a CYB-3 antibody and subjected to western analysis with the same antibody. α-tubulin was used as a loading control. Asterisk: non-specific protein band. B) A Clustal-W alignment of approximately 1000 nucleotides from the N-terminal protein coding region of *cyb-1*, *cyb-2.1*, *cyb-2.2*, and *cyb-3* cDNAs. The percent identity among the four *C. elegans* B-type cyclins is listed in the table below.
(0.87 MB TIF)

Figure S2 Mitotic chromosome condensation is modestly delayed in CYB-3 depleted embryos
Selected images of the male pronucleus from TH32 embryos treated with the indicated RNAi are shown. Time 0:00 =  NEB, intervals are 30 seconds. Images are flattened from five 1 µm optical slices.
(0.73 MB TIF)

Figure S3 Depletion of different *C. elegans* Cyclin B proteins leads to distinct mitotic defects. A) Embryos from OD57 hermaphrodites microinjected with *control*, *cyb-3*, and *cyb-1&2* double-stranded RNA were subjected to live imaging. Time 0:00 corresponds to NEB. Frames to the right of 0:00 depict mitotic progression in minutes after NEB. PNM: Pronuclear meeting; NEB: nuclear envelope breakdown. Scale bar: 10 µm. B) Mitotic progression in *control* and *cyb-3(RNAi)* treated OD57 embryos undergoing the first mitotic division. Error bars: SEM, n = 4 embryos for each condition. Metaphase*: metaphase in *cyb-3(RNAi)* embryos was defined as the interval between near complete chromosome alignment and centrosome breakdown.
(1.21 MB TIF)

Figure S4 Completion of meiosis does not affect the metaphase delay in *cyb-3(RNAi)* embryos
OD57 embryos treated with *control* or *cyb-3(RNAi)* were subjected to live imaging. 0:00 =  NEB. Arrows point to two extruded polar bodies indicating that the MI and MII divisions were complete. Scale bar: 10 µm.
(1.21 MB TIF)

Figure S5 3F3/2 immunostaining of *C. elegans* chromosomes is increased upon taxol exposure
Wild-type embryos treated with vehicle or taxol were fixed and stained as described in Materials and Methods. Individual nuclei are shown. 3F3/2 immunostaining (red) is localized to chromosomes in taxol-treated cells. Scale bar: 1 µm.
(0.45 MB TIF)

Figure S6 DNC-1/p150(glued) is sequestered at chromosomes in CYB-3 depleted cells
*Control* and *cyb-3(RNAi)* embryos were fixed and stained with DAPI (blue) and antibodies recognizing α-tubulin (green) and DNC-1 (red). Arrows: DNC-1 centrosome staining in control embryos that is decreased upon CYB-3 depletion. Arrowhead: centrosome breakdown. Scale bar: 10 µm.
(3.28 MB TIF)

Figure S7 *cyb-3(RNAi)* K-Mts are accessible to microtubule-associated proteins
*Control* and *cyb-3(RNAi)* embryos were fixed and stained with DAPI (blue) and antibodies recognizing α-tubulin (green) and BMK-1 (red). Scale bar: 10 µm.
(1.38 MB TIF)

Text S1 Supplemental Materials and Methods.
(0.04 MB DOC)

Video S1 Meiotic divisions in a fertilized *control(RNAi)* oocyte. A fertilized oocyte from a *control(RNAi)-*treated OD57 hermaphrodite (QuickTime; 1.2 MB; 2.5 frames/sec).
(1.29 MB MOV)

Video S2 Meiotic divisions in a fertilized *cyb-3(RNAi)* oocyte. A fertilized oocyte from a *cyb-3(RNAi)-*treated OD57 hermaphrodite (QuickTime; 2 MB; 2.5 frames/sec).
(2.10 MB MOV)

Video S3 Pronuclear migration in a *control(RNAi)* embryo. A one-cell embryo from a *control(RNAi)-*treated TH32 hermaphrodite (QuickTime; 1.8 MB; 2.5 frames/sec).
(1.83 MB MOV)

Video S4 Pronuclear migration in a *cyb-3(RNAi)* embryo. A one-cell embryo from a *cyb-3(RNAi)-*treated TH32 hermaphrodite (QuickTime; 1.2 MB; 2.5 frames/sec).
(1.31 MB MOV)

Video S5 First mitosis in a *control(RNAi)* embryo. A one-cell embryo from a *control(RNAi)-*treated OD57 hermaphrodite (QuickTime; 743 KB; 2.5 frames/sec).
(0.75 MB MOV)

Video S6 First mitosis in a *cyb-3(RNAi)* embryo. A one-cell embryo from a *cyb-3(RNAi)-*treated OD57 hermaphrodite (QuickTime; 548 KB; 2.5 frames/sec).
(0.56 MB MOV)

Video S7 First mitosis in a *cyb-1&2(RNAi)* embryo. A one-cell embryo from a *cyb-1&2(RNAi)*-treated OD57 hermaphrodite (QuickTime; 472 KB; 2.5 frames/sec).
(0.48 MB MOV)

Video S8 First mitosis in a *cyb-3+control(RNAi)* embryo. A one-cell embryo from a *cyb-3+control(RNAi)-*treated OD57 hermaphrodite (QuickTime; 1 MB; 2.5 frames/sec).
(1.08 MB MOV)

Video S9 First mitosis in a *mdf-1+control(RNAi)* embryo. A one-cell embryo from a *mdf-1+control(RNAi)-*treated OD57 hermaphrodite (QuickTime; 456 KB; 2.5 frames/sec).
(0.47 MB MOV)

Video S10 First mitosis in a *cyb-3+mdf-1(RNAi)* embryo. A one-cell embryo from a *cyb-3+mdf-1(RNAi)-*treated OD57 hermaphrodite (QuickTime; 544 KB; 2.5 frames/sec).
(0.56 MB MOV)

Video S11 First mitosis in a *cyb-3+san-1(RNAi)* embryo. A one-cell embryo from a *cyb-3+san-1(RNAi)-*treated OD57 hermaphrodite (QuickTime; 824 KB; 2.5 frames/sec).
(0.84 MB MOV)

Video S12 First mitosis in a *cyb-3+bub-1(RNAi)* embryo. A one-cell embryo from a *cyb-3+bub-1(RNAi)-*treated OD57 hermaphrodite (QuickTime; 236 KB; 2.5 frames/sec).
(0.24 MB MOV)

Video S13 First mitosis in a *smc-4(RNAi)* embryo. A one-cell embryo from a *smc-4(RNAi)-*treated OD57 hermaphrodite (QuickTime; 420 KB; 2.5 frames/sec).
(0.43 MB MOV)

Video S14 GFP::MDF-2 dynamics in a *control(RNAi)* embryo. A one-cell embryo from a *control(RNAi)*-treated OD110 hermaphrodite. Only the GFP channel is shown. (QuickTime; 208 KB; 2.5 frames/sec).
(0.21 MB MOV)

Video S15 GFP::MDF-2 and chromosome dynamics in a *control(RNAi)* embryo. A one-cell embryo from a *control(RNAi)*-treated OD110 hermaphrodite. Both the GFP and mCherry signals are shown (QuickTime; 200 KB; 2.5 frames/sec).
(0.20 MB MOV)

Video S16 GFP::MDF-2 dynamics in a *cyb-3(RNAi)* embryo. A one-cell embryo from a *cyb-3(RNAi)*-treated OD110 hermaphrodite. Only the GFP channel is shown. (QuickTime; 180 KB; 2.5 frames/sec).
(0.18 MB MOV)

Video S17 GFP::MDF-2 and chromosome dynamics in a *cyb-3(RNAi)* embryo. A one-cell embryo from a *cyb-3(RNAi)*-treated OD110 hermaphrodite. Both the GFP and mCherry signals are shown (QuickTime; 172 KB; 2.5 frames/sec).
(0.17 MB MOV)

Video S18 GFP::MDF-2 dynamics in a *smc-4(RNAi)* embryo. A one-cell embryo from a *smc-4(RNAi)*-treated OD110 hermaphrodite. Only the GFP channel is shown. (QuickTime; 248KB; 2.5 frames/sec).
(0.25 MB MOV)

Video S19 GFP::MDF-2 and chromosome dynamics in a *smc-4(RNAi)* embryo. A one-cell embryo from a *smc-4(RNAi)*-treated OD110 hermaphrodite. Both the GFP and mCherry signals are shown (QuickTime; 244 KB; 2.5 frames/sec).
(0.25 MB MOV)

Video S20 GFP::DHC-1 dynamics in a *control(RNAi)* embryo. A one-cell embryo from a *control(RNAi)*-treated OD203 hermaphrodite (QuickTime; 452 KB; 2.5 frames/sec).
(0.46 MB MOV)

Video S21 GFP::DHC-1 and chromosome dynamics in a *control(RNAi)* embryo. A one-cell embryo from a *control(RNAi)*-treated OD203 hermaphrodite (QuickTime; 440 KB; 2.5 frames/sec).
(0.45 MB MOV)

Video S22 GFP::DHC-1 dynamics in a *smc-4(RNAi)* embryo. A one-cell embryo from a *smc-4(RNAi)*-treated OD203 hermaphrodite (QuickTime; 484 KB; 2.5 frames/sec).
(0.49 MB MOV)

Video S23 GFP::DHC-1 and chromosome dynamics in a *smc-4(RNAi)* embryo. A one-cell embryo from a *smc-4(RNAi)*-treated OD203 hermaphrodite (QuickTime; 504 KB; 2.5 frames/sec).
(0.51 MB MOV)

Video S24 GFP::DHC-1 dynamics in a *mdf-1+control(RNAi)* embryo. A one-cell embryo from a *mdf-1+control(RNAi)*-treated OD203 hermaphrodite (QuickTime; 160 KB; 2.5 frames/sec).
(0.16 MB MOV)

Video S25 GFP::DHC-1 and chromosome dynamics in a *mdf-1+control(RNAi)* embryo. A one-cell embryo from a *mdf-1+control(RNAi)*-treated OD203 hermaphrodite (QuickTime; 164 KB; 2.5 frames/sec).
(0.17 MB MOV)

Video S26 GFP::DHC-1 dynamics in a *cyb-3*+*control(RNAi)* embryo. A one-cell embryo from a *cyb-3+control(RNAi)*-treated OD203 hermaphrodite (QuickTime; 504 KB; 2.5 frames/sec).
(0.51 MB MOV)

Video S27 GFP::DHC-1 and chromosome dynamics in a *cyb-3*+*control(RNAi)* embryo. A one-cell embryo from a *cyb-3+control(RNAi)*-treated OD203 hermaphrodite (QuickTime; 480 KB; 2.5 frames/sec).
(0.49 MB MOV)

Video S28 GFP::DHC-1 dynamics in a *cyb-3+mdf-1(RNAi)* embryo. A one-cell embryo from a *cyb-3+mdf-1(RNAi)*-treated OD203 hermaphrodite (QuickTime; 840 KB; 2.5 frames/sec).
(0.86 MB MOV)

Video S29 GFP::DHC-1 and chromosome dynamics in a *cyb-3+mdf-1(RNAi)* embryo. A one-cell embryo from a *cyb-3+mdf-1(RNAi*)-treated OD203 hermaphrodite (QuickTime; 820 KB; 2.5 frames/sec).
(0.84 MB MOV)

Video S30 First mitosis in a *dilute cyb-3(RNAi)* embryo. A one-cell embryo from a *dilute cyb-3(RNAi)-*treated OD57 hermaphrodite (QuickTime; 312 KB; 2.5 frames/sec).
(0.32 MB MOV)

Video S31 First mitosis in a *dilute cyb-3+dylt-1(RNAi)* embryo. A one-cell embryo from a *dilute cyb-3+dylt-1(RNAi)-*treated OD57 hermaphrodite (QuickTime; 296 KB; 2.5 frames/sec).
(0.30 MB MOV)

Video S32 First mitosis in a *dylt-1(RNAi)* embryo. A one-cell embryo from a *dylt-1(RNAi)-*treated OD57 hermaphrodite (QuickTime; 436 KB; 2.5 frames/sec).
(0.44 MB MOV)
