# Supplementary figures and images for: Caenorhabditis elegans Cyclin B3 Is Required for Multiple Mitotic Processes Including Alleviation of a Spindle Checkpoint–Dependent Block in Anaphase Chromosome Segregation
Source: PLoS Genet. 2010 Nov 24;6(11):e1001218. doi: 10.1371/journal.pgen.1001218 (PMC2991249; doi:10.1371/journal.pgen.1001218)

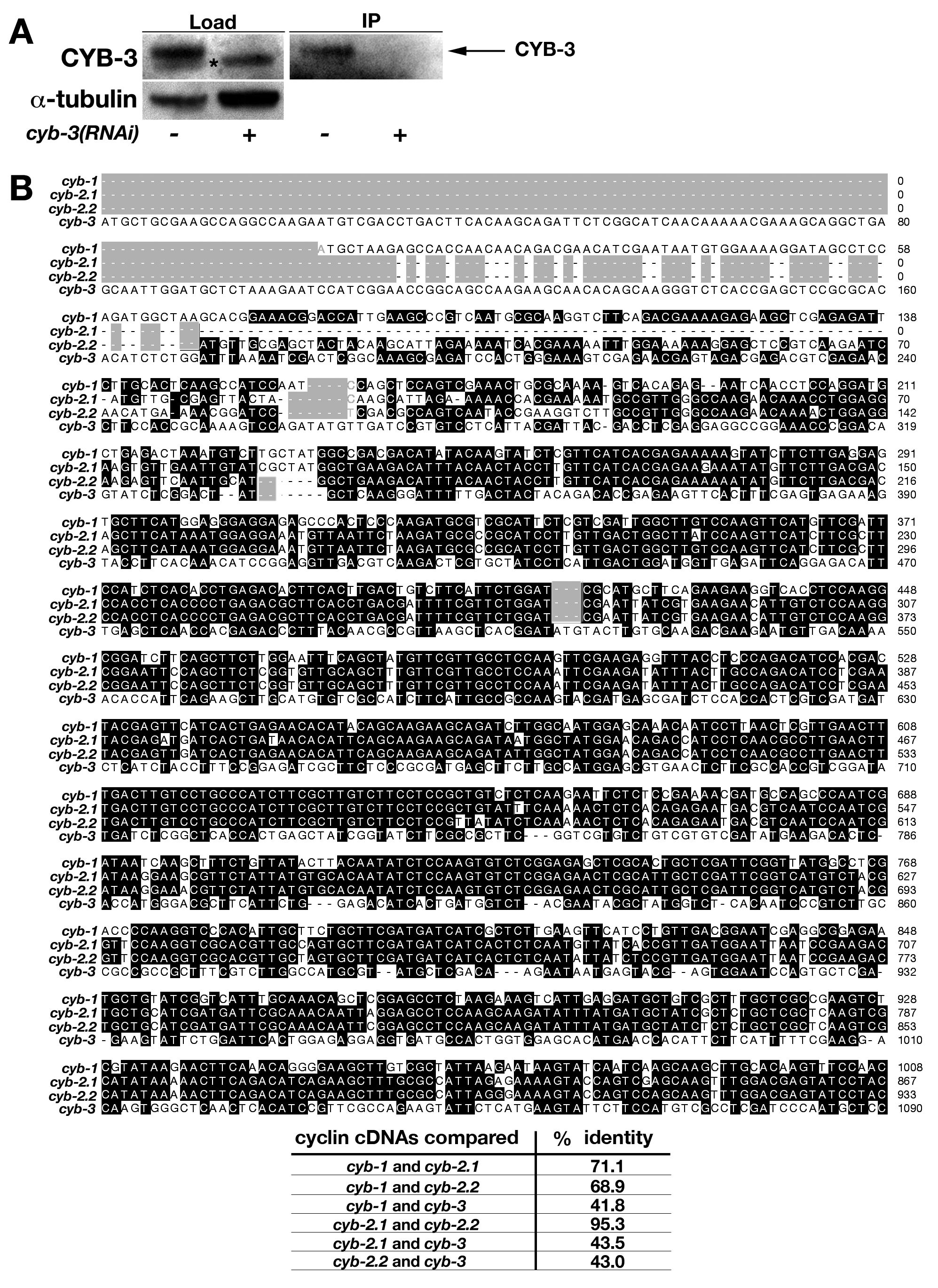

Supplement: Figure S1 — cyb-3(RNAi) efficiency and an alignment of C. elegans B-type cyclins. A) Protein extracts from control and cyb-3(RNAi) embryos were immunoprecipitated with a CYB-3 antibody and subjected to western analysis with the same antibody. α-tubulin was used as a loading control. Asterisk: non-specific protein band. B) A Clustal-W alignment of approximately 1000 nucleotides from the N-terminal protein coding region of cyb-1, cyb-2.1, cyb-2.2, and cyb-3 cDNAs. The percent identity among the four C. elegans B-type cyclins is listed in the table below. (0.87 MB TIF) [file pgen.1001218.s001.tif]

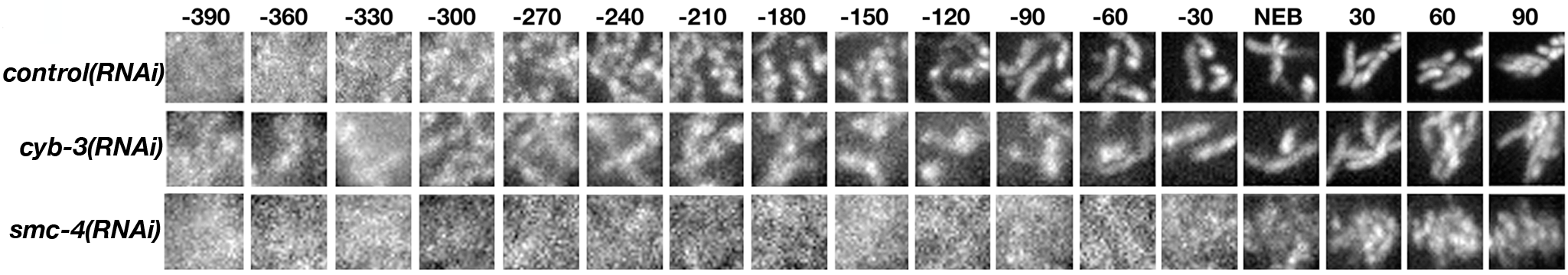

Supplement: Figure S2 — Mitotic chromosome condensation is modestly delayed in CYB-3 depleted embryos Selected images of the male pronucleus from TH32 embryos treated with the indicated RNAi are shown. Time 0:00 = NEB, intervals are 30 seconds. Images are flattened from five 1 µm optical slices. (0.73 MB TIF) [file pgen.1001218.s002.tif]

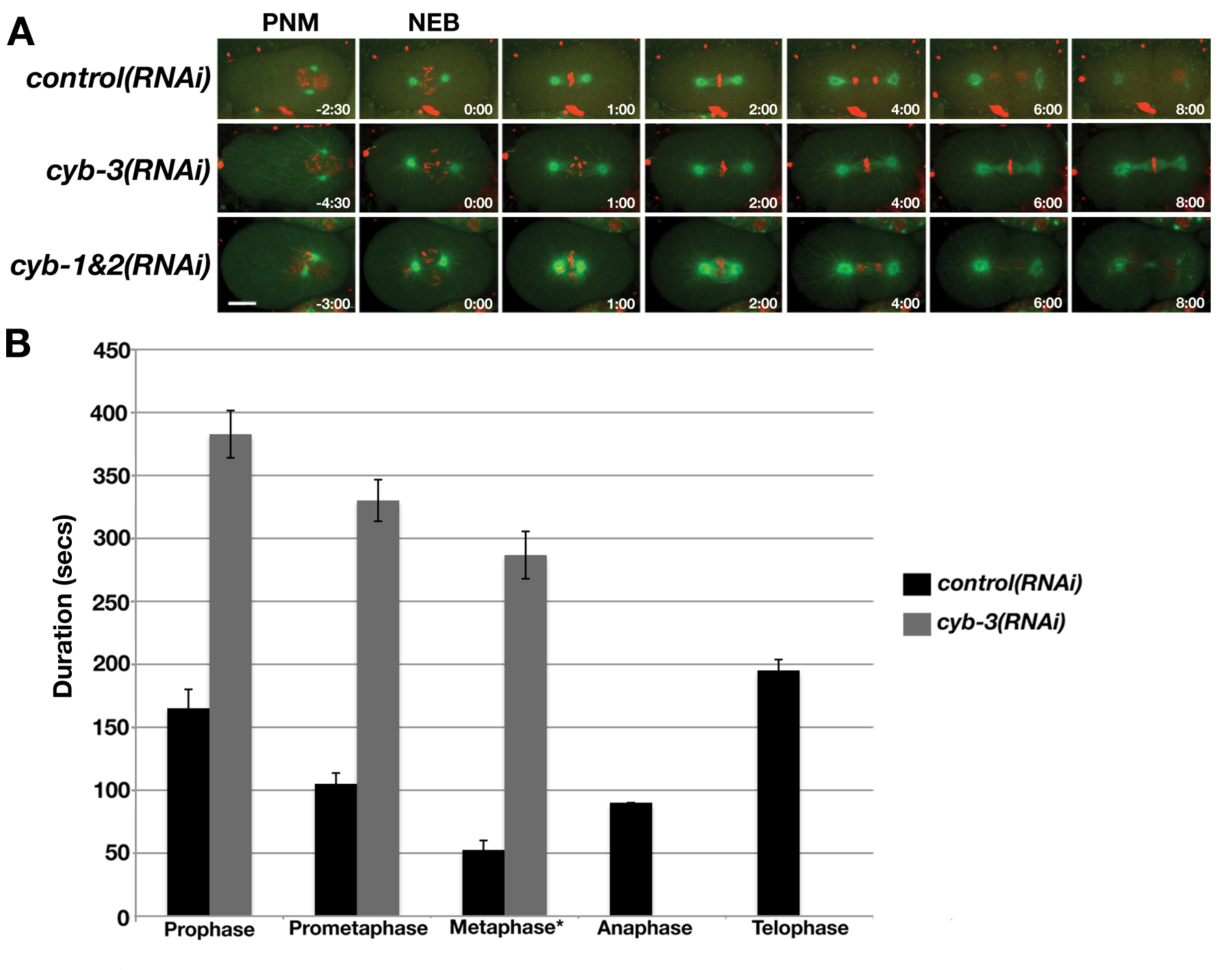

Supplement: Figure S3 — Depletion of different C. elegans Cyclin B proteins leads to distinct mitotic defects. A) Embryos from OD57 hermaphrodites microinjected with control, cyb-3, and cyb-1&2 double-stranded RNA were subjected to live imaging. Time 0:00 corresponds to NEB. Frames to the right of 0:00 depict mitotic progression in minutes after NEB. PNM: Pronuclear meeting; NEB: nuclear envelope breakdown. Scale bar: 10 µm. B) Mitotic progression in control and cyb-3(RNAi) treated OD57 embryos undergoing the first mitotic division. Error bars: SEM, n = 4 embryos for each condition. Metaphase*: metaphase in cyb-3(RNAi) embryos was defined as the interval between near complete chromosome alignment and centrosome breakdown. (1.21 MB TIF) [file pgen.1001218.s003.tif]

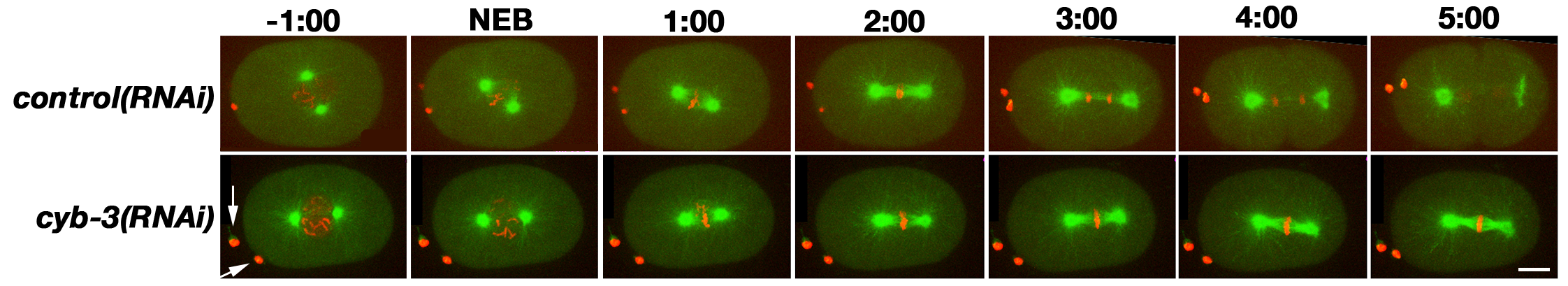

Supplement: Figure S4 — Completion of meiosis does not affect the metaphase delay in cyb-3(RNAi) embryos OD57 embryos treated with control or cyb-3(RNAi) were subjected to live imaging. 0:00 = NEB. Arrows point to two extruded polar bodies indicating that the MI and MII divisions were complete. Scale bar: 10 µm. (1.21 MB TIF) [file pgen.1001218.s004.tif]

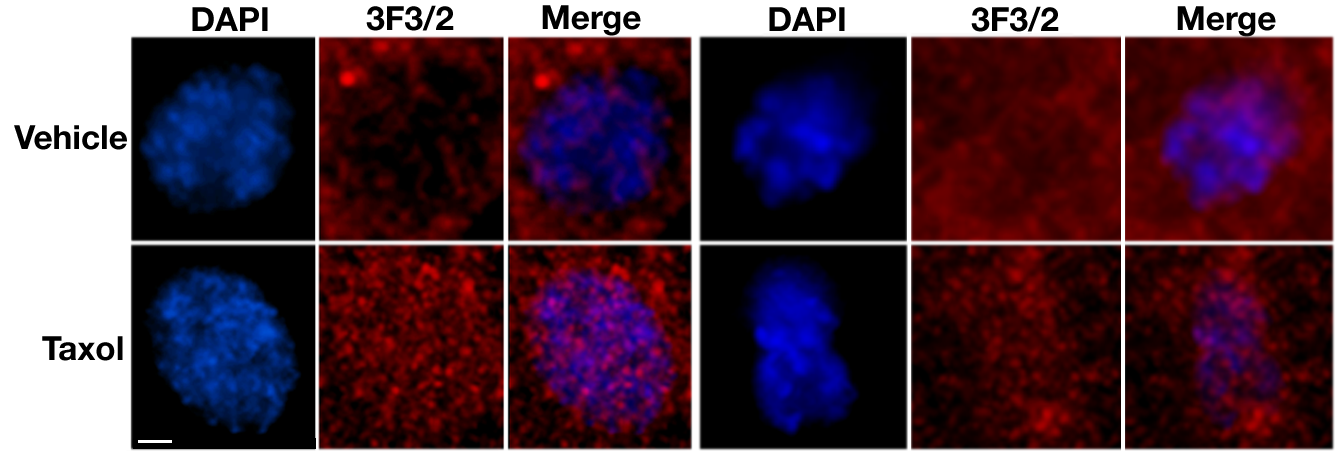

Supplement: Figure S5 — 3F3/2 immunostaining of C. elegans chromosomes is increased upon taxol exposure Wild-type embryos treated with vehicle or taxol were fixed and stained as described in Materials and Methods. Individual nuclei are shown. 3F3/2 immunostaining (red) is localized to chromosomes in taxol-treated cells. Scale bar: 1 µm. (0.45 MB TIF) [file pgen.1001218.s005.tif]

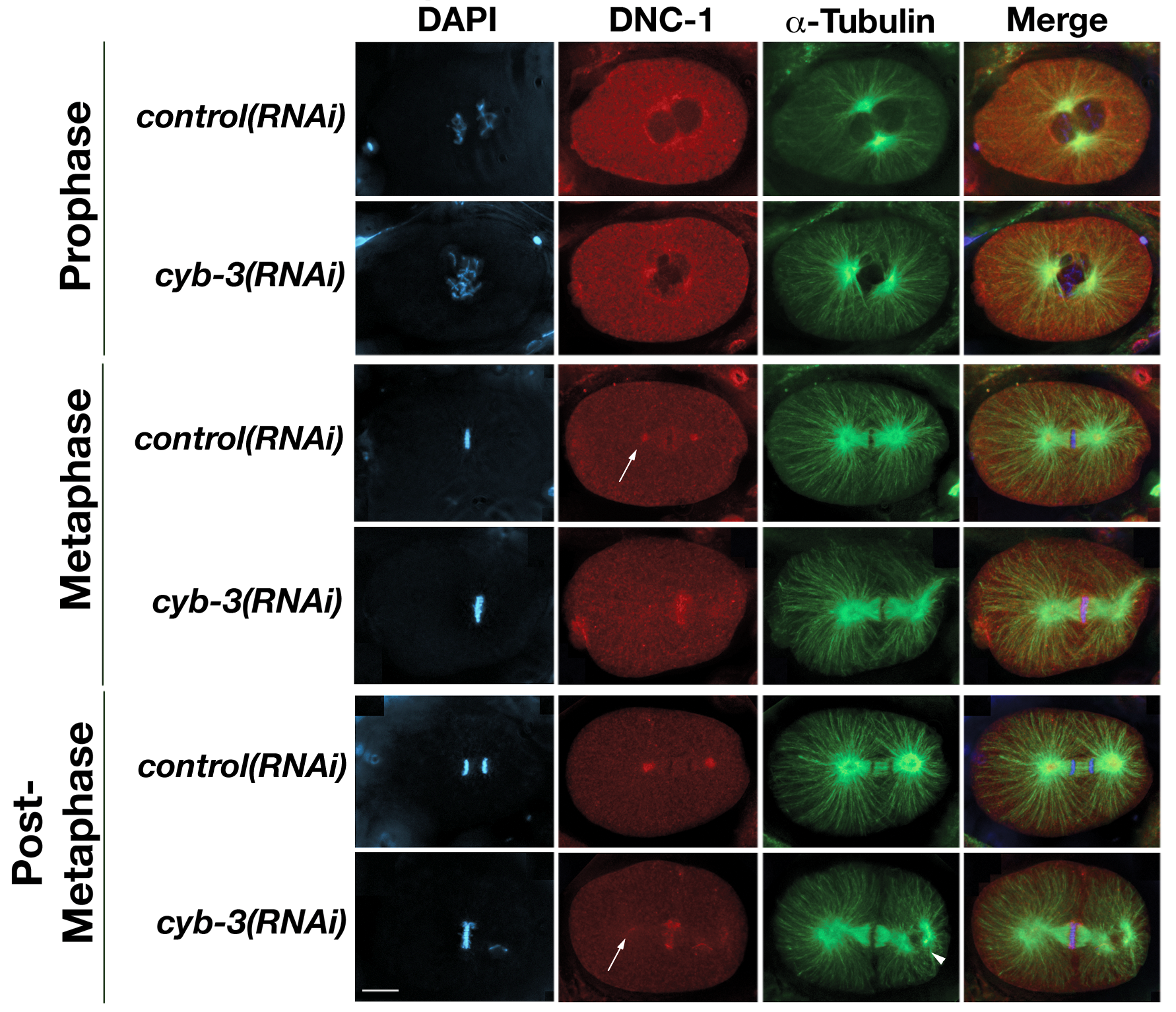

Supplement: Figure S6 — DNC-1/p150(glued) is sequestered at chromosomes in CYB-3 depleted cells Control and cyb-3(RNAi) embryos were fixed and stained with DAPI (blue) and antibodies recognizing α-tubulin (green) and DNC-1 (red). Arrows: DNC-1 centrosome staining in control embryos that is decreased upon CYB-3 depletion. Arrowhead: centrosome breakdown. Scale bar: 10 µm. (3.28 MB TIF) [file pgen.1001218.s006.tif]

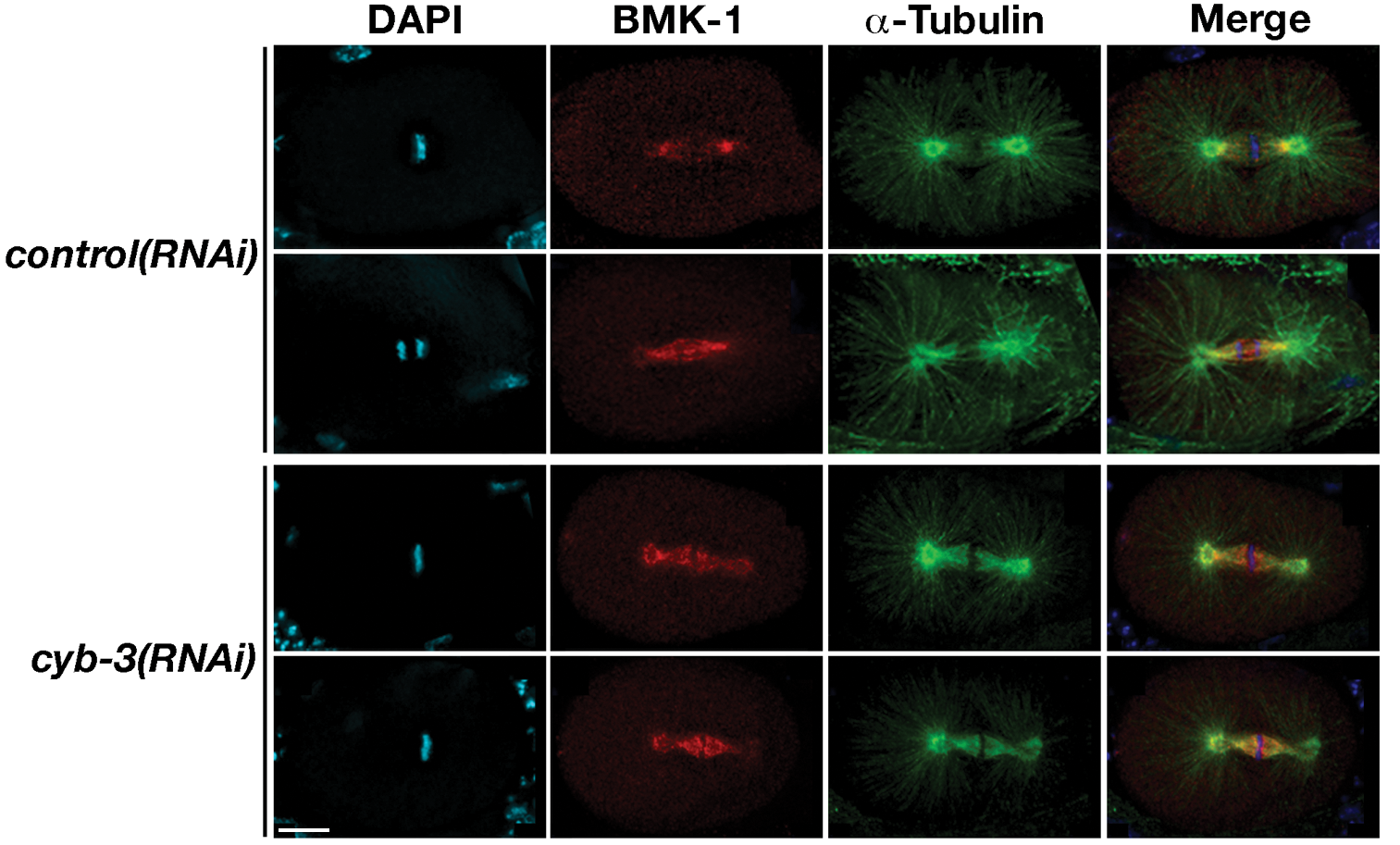

Supplement: Figure S7 — cyb-3(RNAi) K-Mts are accessible to microtubule-associated proteins Control and cyb-3(RNAi) embryos were fixed and stained with DAPI (blue) and antibodies recognizing α-tubulin (green) and BMK-1 (red). Scale bar: 10 µm. (1.38 MB TIF) [file pgen.1001218.s007.tif]
