# Supplementary material for: Caenorhabditis elegans Cyclin B3 Is Required for Multiple Mitotic Processes Including Alleviation of a Spindle Checkpoint–Dependent Block in Anaphase Chromosome Segregation
Source: PLoS Genet. 2010 Nov 24;6(11):e1001218. doi: 10.1371/journal.pgen.1001218 (PMC2991249; doi:10.1371/journal.pgen.1001218)
Supplement: Text S1 — Supplemental Materials and Methods. (0.04 MB DOC) [file pgen.1001218.s008.doc]

**SUPPLEMENTAL MATERIALS AND METHODS**

**Immunoprecipitation and western analysis**

Gravid N2 *C. elegans* hermaphrodites treated with control or *cyb-3(RNAi)* were subjected to the alkaline hypochlorite method to isolate embryos [1].  Embryos were briefly washed in PBS and resuspended in lysis buffer (PBS, 20mM HEPES, 1% NP-40, 50 μM β-glycerophosphate, 1 mM Na_3_VO_4_, 1 mM dithiothreitol [DTT], 1 mM EDTA, 1 mM PMSF + complete protease inhibitors (Roche Diagnostics, Indianapolis, IN)) and sonicated 3 times over ice for 30 seconds each. Following centrifugation at 12,000 rpm for 10 minutes, the clarified lysates were immediately used for immunoprecipitation.  A 1/10 volume of Protein G-Sepharose beads (GE Healthcare, Piscataway, NJ) was added to the lysates and rocked at 4°C for 1 hour to preclear the extracts. The supernatant was isolated after a brief low-speed spin to pellet the beads.  Protein concentration was determined by Bradford assay (Bio-Rad, Hercules, CA).

For immunoprecipitations, 500 embryos μg embryo extract was incubated with 3 μl CYB-3 antibody [2] O/N at 4°C.  50 μl protein G-Sepharose beads (in a 50:50 slurry) were added and the extract incubated at 4°C for an additional 2 hours.  The beads and isolated immunocomplexes were pelleted via low-speed centrifugation and washed four times in lysis buffer without NP-40. Samples were separated by SDS-PAGE, transferred to nitrocellulose, and the membranes probed with CYB-3 antibody (1:1000) or α-tubulin antibody (1:3000). Western analysis was performed as previously described [1].

**Taxol experiments**

For taxol treatment, approximately 30 gravid adult hermaphrodites were placed in a 30 μl drop of 1:9 bleach solution (diluted from a 6.15% sodium hypochlorite stock with M9) on a microscope slide at RT. The animals were cut open to release embryos, and after a 3 minute incubation, embryos were collected in 1 ml M9 and centrifuged at 1,500 rpm for 1 minute.  The embryo pellet was resuspended in 30 μl chitinase (1 mg/ml), incubated for 5 minutes, and pelleted at 10,000 rpm for 20 seconds.  Pellets were resuspended in 10 μl taxol (100 mM in (M9 + 1% Ethanol); Oregon Green 488 Paclitaxel, Molecular Probes, Eugene, OR) or vehicle (M9 + 1% Ethanol) and incubated on a microscope slide for 4 minutes at RT.  A coverslip was placed on the slide and slight pressure was applied followed by a 1 minute incubation to facilitate taxol entry. Slides were then placed on a metal plate chilled with dry ice and immunostained as above.

**SUPPLEMENTAL REFERENCES**

1. Schumacher JM, Golden A, Donovan PJ (1998) AIR-2: An Aurora/Ipl1-related protein kinase associated with chromosomes and midbody microtubules is required for polar body extrusion and cytokinesis in Caenorhabditis elegans embryos. J Cell Biol 143: 1635-1646.

2. Sonneville R, Gonczy P (2004) Zyg-11 and cul-2 regulate progression through meiosis II and polarity establishment in C. elegans. Development 131: 3527-3543.
